# X-ray Structure Characterization of the Selective Recognition of AT Base Pair Sequences

**DOI:** 10.1021/acsbiomedchemau.3c00002

**Published:** 2023-04-05

**Authors:** Edwin N. Ogbonna, Ananya Paul, Abdelbasset A. Farahat, J. Ross Terrell, Ekaterina Mineva, Victor Ogbonna, David W Boykin, W. David Wilson

**Affiliations:** † Department of Chemistry and Center for Diagnostics and Therapeutics, Georgia State University, Atlanta, Georgia 30303-3083, United States; ‡ Department of Pharmaceutical Organic Chemistry, Faculty of Pharmacy, Mansoura University, Mansoura 35516, Egypt; § Master of Pharmaceutical Sciences Program, California North State University, 9700 W Taron Dr., Elk Grove, California 95757, United States

**Keywords:** X-ray crystal, AT minor groove binder, heterocyclic amidines, sequence specificity, molecular dynamic simulations, water interactions in the DNA minor groove, water-mediated H-bond

## Abstract

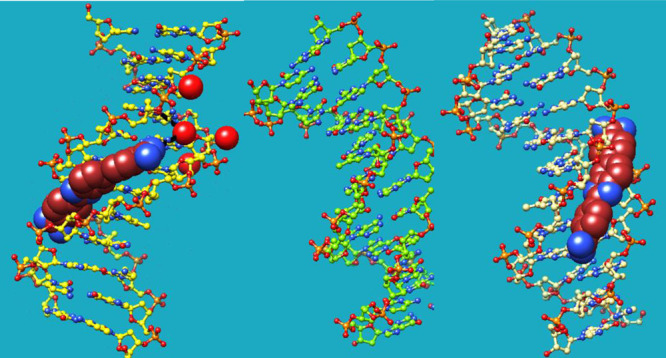

The rational design of small molecules that target specific DNA sequences is a promising strategy to modulate gene expression. This report focuses on a diamidinobenzimidazole compound, whose selective binding to the minor groove of AT DNA sequences holds broad significance in the molecular recognition of AT-rich human promoter sequences. The objective of this study is to provide a more detailed and systematized understanding, at an atomic level, of the molecular recognition mechanism of different AT-specific sequences by a rationally designed minor groove binder. The specialized method of X-ray crystallography was utilized to investigate how the sequence-dependent recognition properties in general, A-tract, and alternating AT sequences affect the binding of diamidinobenzimidazole in the DNA minor groove. While general and A-tract AT sequences give a narrower minor groove, the alternating AT sequences intrinsically have a wider minor groove which typically constricts upon binding. A strong and direct hydrogen bond between the N-H of the benzimidazole and an H-bond acceptor atom in the minor groove is essential for DNA recognition in all sequences described. In addition, the diamidine compound specifically utilizes an interfacial water molecule for its DNA binding. DNA complexes of AATT and AAAAAA recognition sites show that the diamidine compound can bind in two possible orientations with a preference for water-assisted hydrogen bonding at either cationic end. The complex structures of AAATTT, ATAT, ATATAT, and AAAA are bound in a singular orientation. Analysis of the helical parameters shows a minor groove expansion of about 1 Å across all the nonalternating DNA complexes. The results from this systematic approach will convey a greater understanding of the specific recognition of a diverse array of AT-rich sequences by small molecules and more insight into the design of small molecules with enhanced specificity to AT and mixed DNA sequences.

## 1 Introduction

Minor groove-binding drugs have been primarily AT-specific agents such as netropsin, pentamidine, Hoechst dyes, DAPI, Ridinilazole, and many related compounds. Such compounds have a wide range of applications in biotechnology and therapeutics.^1−9^ Drugs, including those from natural products, that target the major groove are also of interest and have many possible applications.^10,11^ Unfortunately, it has proven much more difficult to selectively target the major groove, and the minor groove has been the target of most research to date. Also, targeting the minor groove of double-helical structures of RNA with selective compounds is of major interest;^12^ however, selective targeting of the RNA minor groove with small molecules can be challenging because of the shape of its helical dsRNA.^13−16^

**Figure 1:**
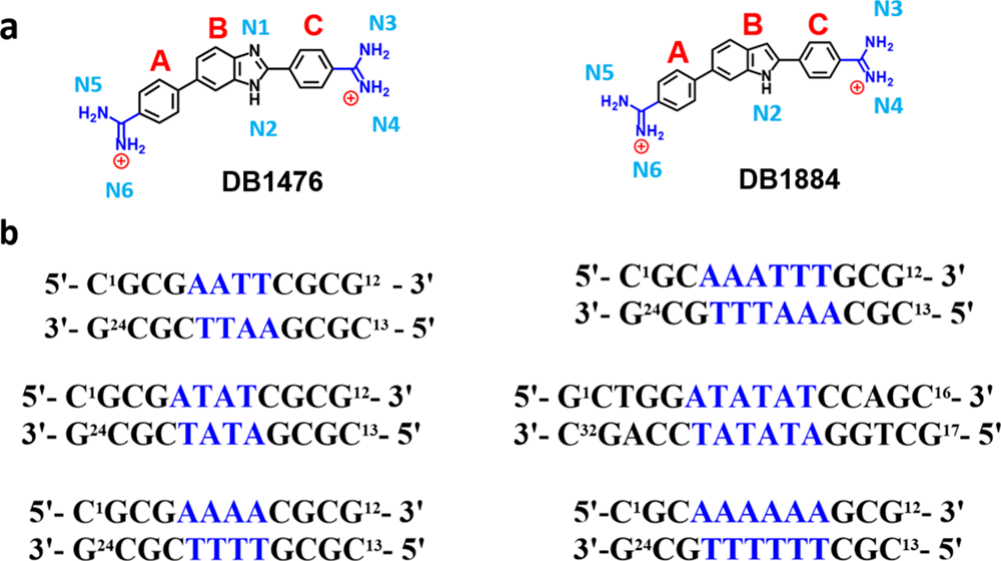
(a) Chemical structure of the DB1476 and DB1884 used in these studies: Benzimidazole and amidines −Ns– have been numbered for result analysis. (b) DNA sequences used for X-ray crystallography and MD analysis.

Much of our laboratory’s recent work has focused on enhancing the applications of minor groove compounds by increasing their sequence recognition capacity to include GC base pairs (bps).^17−22^ An essential compound in this process is a benzimidazole-diphenyl diamidine, DB1476 (Figure 1), which is a relatively small but strong binding AT-specific compound.^23^ A minor modification of DB1476, the conversion of the inner facing −CH of the benzimidazole to nitrogen, converted the compound from an AT-specific to a slightly GC-selective agent. Additional modifications further converted it to one of our most GC-specific compounds.^23^ As we carried out these selective design studies, we realized that there have been relatively few systematic studies on the variations in minor groove-binding to different AT base pair sequences. The importance of therapeutic targeting of AT sites has taken on increased significance with the discovery that minor groove-binding heterocyclic diamidines could inhibit the PU.1 transcription factor by binding upstream AT-rich regions that are nearly ubiquitous in its promoter sites.^6,24^ With our small-molecule inhibitors, we found that PU.1 inhibition is effective at disrupting AML cell growth in human cell lines and primary AML patients’ cells in vitro and in vivo and is a new strategy for the treatment of AML.^6^

In this report, we focus on the structural comparison of the interactions of DB1476 with a variety of AT sequences. Although the isomeric structures −AATT– and −ATAT– are known to be significantly different, for example, in minor groove width,^25,26^ there have been very few systematic crystallographic structural comparisons of minor groove binders with these or other AT sequences.^27−29^ The structures of the two DNAs have been solved with bound netropsin by different groups.^30−33^ In the initial structure of netropsin with d(5′-CGCGAATTCGCG-3′)_2_ by Dickerson and co-workers, a unique conformation of the polyamide in the AATT site was observed. The binding of the compound displaced the ordered minor groove water from –AATT−, which is characteristic of the narrow minor groove in A-tract sites.^30^ In this case and with other complexes with a narrow minor groove, a high propeller twist was observed and led to the conclusion that the high propeller twist and the narrow groove are correlated.

Here, we report the interaction of the compound with DNA sequences containing the four AT binding sites: AATT and ATAT in self-complementary duplex structures. We have also investigated a longer AAATTT and ATATAT sequence for a more extended binding site reference as well as −AAAA– and −AAAAAA–. For each sequence, we report the compound-DNA binding affinities determined by biosensor-SPR methods, the crystal structures of the complexes, and the results of molecular dynamics (MD) simulations of DB1476 with the −AATT– site. Some surprising differences in DB1476 binding among these sequences have been observed. For comparison, the structure of the indole derivative of DB1476, DB1884, with −AATT– was also determined.

## 2 Materials and Methods

### 2.1 Compound Synthesis

For the detailed synthesis scheme of 2,5-diamidinobenzimidazole (DB1476) and 2,6-diphenylindole diamidine (DB1884), see Farahat et al.^34^

### 2.2 Crystallography

The oligonucleotide duplexes d(5′-CGCGAATTCGCG-3′)_2_, d(5′-CGCGATATCGCG-3′)_2_, d(5′-CGCAAATTTGCG-3′)_2_, d(5′-CGCGAAAACGCG-3′/5′-CGCGTTTTCGCG-3′) d(5′-CGCAAAAAAGCG-3′/5′-CGCTTTTTTGCG-3′) from Integrated DNA Technologies, Coralville, IA, were annealed at 85 °C for 6 mins in 20 mM Tris HCl pH 7.4 with 1.5–2.5 stoichiometric equivalents of compound before crystallization. Ligand-bound and native d(5′-GCTGGATATATCCAGC-3′)_2_ (Integrated DNA Technologies, Coralville, IA) duplexes were annealed at 1 mM concentration at 85 °C for 6 mins in 7.5 mM HEPES pH 6.6 in the presence and absence of three stochiometric equivalents of DB1476. All dodecamer (12-mer) crystals were grown by vapor diffusion in 4 μL hanging drops (1:1) at 298 K in 24-well VDX plates (Hampton Research) in drops containing the 24-conditions of the Nucleic Acid Mini-screen (Hampton Research) against wells containing 600 μL of a 35% solution of (±)-2-methyl-2,4-pentanediol (MPD). d(5′-CGCGAATTCGCG-3′)_2_ −DB1476 complex crystals were grown using condition 9 in a drop composed of 20 mM MgCl_2_. 6H_2_O, 80 mM NaCl, 12 mM KCl, 1.00 mM double-stranded DNA, 1.6 mM DB1476, 10% v/v (±)-2-methyl-2,4-pentanediol (MPD), 12 mM spermine tetrahydrate, and 40 mM sodium cacodylate trihydrate buffer at pH 6.0. Rod-shaped colorless crystals were observed within 3 weeks.

Both ligand-bound and unbound d(5′-GCTGGATATATCCAGC-3′)_2_ (16-mer) crystals were obtained in 6 μL hanging drops comprised of a 1:1 mixture with well solution containing 600 mM CaCl_2_, 10 mM HEPES pH 8.6, and 40% PEG200 with crystals appearing overnight. See supplemental information (SI) Table S1 for the crystallization details of the remaining crystals used for this study. Before data collection, all crystals were prepared by transferring them to appropriately sized cryo-loops and flash-frozen in liquid nitrogen.

X-ray diffraction data sets were collected at SER-CAT at the Argonne National Laboratory Advanced Photon Source (APS) (Lemont, IL), Berkeley National Laboratory Advanced Light Source (ALS) (Berkeley, CA), and the National Synchrotron Light Source II (NSLS-II) at Brookhaven National Laboratory (BNL) (Upton, NY). See Table S2 for crystal sample collection sources and details.

### 2.3 Structure Solution and Refinement

Crystallographic indexing, integration, and scaling were done via autoprocessing in XDS. Data reduction was carried out using Aimless in the Collaborative Computative Project No.4 2 (CCP4i2) software suite.^35^ All crystal structures were solved by molecular replacement using maximum-likelihood search procedures in PHASER-MR and refined using Phenix.refine in the PHENIX suite.^36^ Structure solution and refinement were carried out using an established DNA model (PDB entry 1BNA).

Refinement strategies included rigid body and restrained refinements. The addition of ligands and water using the Crystallographic Object-Oriented Toolkit (COOT) software^37^ followed by additional refinements to the models, resulted in their final *R* values. For the scope of this study, it is important to emphasize the significance of *R* values.

*R* values are standardized concepts in crystallography that measure how accurate the refined models are to experimental data. Thus, *R* values measure how well the calculated diffraction pattern matches the experimentally observed pattern. *R*_work_ and *R*_free_ are collectively known as *R* values. Previously, *R*_work_ (also *R* factor) was the sole *R* value until the introduction of *R*_free_ to eliminate the introduction of bias during refinement. Therefore, every *R*_work_ value has a corresponding *R*_free_ value, and *R*_free_ has become the primary *R* value used to measure model accuracy. For a structure model that agrees well with experimental data, the value of *R*_work_ should always be less than *R*_free_ and their difference should be no greater than 0.05. Most structures with a resolution of 2 Å or better will have an *R* value <0.26. Structures with a resolution >2 Å whose *R* values significantly exceed 0.3 suggest there may be errors in the model.

The crystallographic statistics of data collection and refinements can be found in Table S3 SI. The 2*F*_o_–*F*_c_ maps for all X-ray structures determined in this study show substantial electron density occupancy with their ligands and global structures. The atomic structure and coordinate factors for DNA and DNA-ligand complexes have been deposited to the RCSB Protein and Nucleic Acid Data Bank. Chimera X software generated all the figures containing crystal structures and models.^38^ The DNA properties and helical parameters were analyzed for studies using the Chimera X and 3DNA software.^39^

### 2.4 Ab Initio Calculations and MD Simulations

Geometry optimization and electrostatic potential calculations for the DB1476 and (Figure 1) were performed by using DFT/B3LYP^40^ functional theory and the 6-31+G* basis set^41^ in Gaussian 09^42^ (Gaussian, Inc., 2009, Wallingford, CT) with Gauss-view. RESP charges for the ligand were calculated by using the Merz–Singh–Kollman scheme.^43,44^ The AMBER16 software suite was used to perform MD simulations.

Canonical *B*-form DNA, *d*[(5′-CGCGAATTCGCG-3′)(5′-CGCGAATTCGCG-3′)] was built in the Nucleic Acid Builder (NAB) tool in AMBER16.^45,46^ AMBER topology files and force field parameter files for DB1476 and DB1884 are required to run MD simulations. These parameters were produced using ANTECHAMBER.^47,48^ Specific atom types assigned for molecules were adapted from the ff99 force field. For diamidine molecules, most of the force field parameters were derived from the existing set of bonds, angles, and dihedrals for similar atom types in the parm99 and GAFF force fields.^49^ Amidine dihedral angle parameters were obtained from previously reported parametrized data.^50,51^

The AutoDock tools^52^ program was used to dock each ligand in the minor groove of the AATT DNA sequence initial structure for DNA-ligand complexes. The X-ray crystal structure of each complex was also used for the initial structure for MD simulation studies. The AMBER16 package was used to equilibrate the ligand–DNA complex system using OL15 force field modifications for DNA.

MD simulations were performed in explicit solvation conditions where DNA and DNA-ligand complexes were solvated in a 72 Å × 72 Å × 72 Å truncated octahedron box filled with approximately 5000 TIP3P water^53^ molecules by using the *Tleap*(43) program in AMBER16. 150 mM Na+ and Cl– ions were added to the systems to reach a physiological salt concentration. 150 mM NaCl is beyond the excess of the Na+ ion necessary to achieve electrical neutrality but is more biologically relevant. The particle mesh Ewald (PME)^54^ method was used to handle Coulombic interactions, and a 10 Å cutoff was applied to all van der Waals interactions. The MD simulations were carried out using the Sander module with the SHAKE^55^ algorithm applied to constrain all bonds involving hydrogen atoms with an integration time step of 2 fs. The system was relaxed in the multistage equilibration protocol with 500 steps of steepest descent energy minimization. The temperature of the system was then increased from 0 to 310 K for over 10 ps under constant-volume conditions. In the final step, the production runs on the system were subsequently performed for 600 ns under NPT (constant-pressure) conditions on the PMEMD CUDA module of AMBER16.^44,46^ Trajectories were postprocessed using the CPPTRAJ module of AMBERTOOLS16^44,46^ to produce 25,000 snapshots for analysis and visualization in UCSF Chimera visualization software.^56^ The steepest descent algorithm is good for quickly removing the largest strains in the system, but it also converges slowly when close to a minimum.

#### 2.4.1 MD Trajectory Analysis

Analysis of the trajectories was performed using cpptraj from AmberTools16.^45,46^ Visualization and data analysis (Bond angles and dihedral angles, bonding distances, throughout the MD simulations) were performed with UCSF Chimera.^56^ The final 500 ns (25,000 frames) were used for analysis.

## 3 Results

### 3.1 X-ray Complex Structure of AATT-DB1476 and Its Minor Groove Interactions

**Figure 2:**
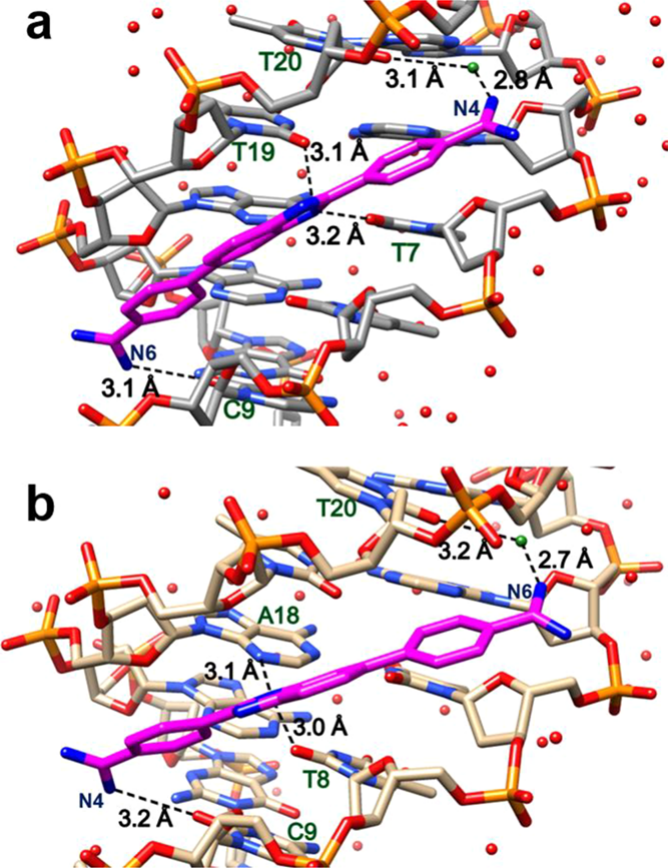
(a) Significant bonding distances in the **AATT-DB1476-I** complex. DNA in gray and DB1476 in magenta. A bifurcated hydrogen bonding between B-N2 (blue) with O2 (red) of T7 and T19 is 3.2 Å and 3.1 Å bonding distance (black dashed lines), respectively. The direct hydrogen bond distance with O2 of C9 is 3.2 Å. Interfacial water-mediated (green, black lines) H-bond distance (N4---O-H_2_) is 2.8 Å and O-H_2_---T20 is 3.1 Å. (b) Significant bonding distances in the **AATT-DB1476-II** complex. DNA in tan and DB1476 in magenta. The bifurcated hydrogen bonding distances between B-N2 (blue) with O2 (red) of T8 and N3 of A18 are 3.0 and 3.1 Å, respectively. The direct hydrogen bond distance with N4 and O2 of C9 is 3.2 Å. The interfacial water-mediated mediated (green, black lines) bonding from N6---O-H_2_ is 2.7 Å and from O-H_2_---T20 is 3.2 Å.

**Figure 3:**
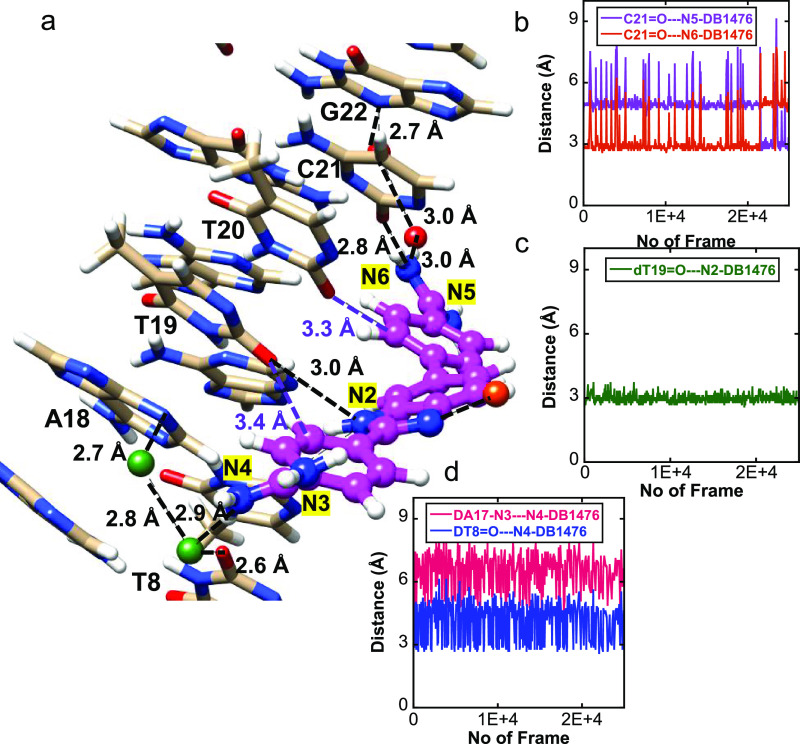
(a) Snapshot of MD simulations of the DB1476-d(CGCGAATTCGCG)_2_ complex; DB1476 bound to the −AATT– site with interfacial water(s)-mediated interaction between the amidines of DB1476 and DNA. The interfacial water (green, ball, stick, and black dashed lines) completes the bound compound’s curvature. DB1476 forms bifurcated direct BI-N-H hydrogen bonds (T19, T7 = O---N2, black dashed lines) and two C-H interactions (purple dashed lines) with DNA bases. Terminal amidine groups also form extended terminal water (red) networks to stabilize the compound in the minor groove. The important distances between different sections of the DB1476-DNA complex are illustrated in (b–d); (b) N5/N6 forms strong H-bonds with O2 of C21 (orange and purple line). The initial frames (1–22,000) represent that N6 is inside the groove and form direct (2.6 to 3.4 Å) or interfacial water-mediated H-bonds (3.6 to 5.8 Å, spike) with O2 of C21 (orange). The rest of the frames represent the 180^o^ rotation of N6, and N5 forms H-bonds with O2 of C21 (purple). (c) Distance plots between the BI-N2 and O2 of T19 (green line). (d) Distance plots of another terminal amidine N3/N4 with O2 of T8 (blue line) and N3 of A17. As described in (a), the bond distances between 2.6 Å and 3.4 Å represent direct H-bonds, and distances with 3.6 Å to 5.8 Å represent interfacial water-mediated H-bonds.

Herein, we report the high-resolution X-ray crystal structures of multiple AT oligomer duplex DNAs with their small-molecule complexes. DB1476, DB1884, and the AT DNA sequences for this study are listed in Figure 1. The AATT-DB1476 complex structure reveals a unique 1:1 binding mode with two orientations of DB1476 observed in the minor groove (Figure 2). The electron density map of AATT-DB1476-I and AATT-DB1476-II does not show an unequivocal orientation assignment. Thus, we presented a structure with two possible ligand orientations (Figure 2). The ligand in the structures AATT-DB1476-I and AATT-DB1476-II (Figure 2) utilizes an interfacial water molecule at one amidine end to bind to the DNA. Model refinements and computational studies have shown the interfacial water to be very dynamic. (Figure 3) The interfacial water dynamics cause the amidine of DB1476 to interact at varying distances with the DNA causing a broadening of the electron density map at that amidine end. The dynamics of the interfacial water and broadening of the electron density at one amidine end can be clearly seen in Figure 3 and Figure S1. The 2*F*_o_–*F*_c_ map for DB1476 in both orientations (Figure S1) shows a good overall structural fit of their electron density map except at the amidine end with the dynamic interfacial water. The statistics of AATT-DB1476-I (PDB ID: 8EC1) and AATT-DB1476-II (PDB ID: 8ED6) are similar with a resolution of 1.63 Å with *R*_free_ value and average B-factor at 22.7%, 22.2% and 27.3, 27.2, respectively (Table S3). Likewise, for A6-DB1476, we also presented a structure with two possible ligand orientations. The significant difference between AATT-DB1476-I and AATT-DB1476-II is the flip of the amidine ends to an opposite orientation (Figure 2).

Minor groove interactions with benzimidazole (BI) diamidines show strong direct hydrogen bonds by the N-H of benzimidazole and amidine groups to the acceptor atoms, O2 of thymine and N3 of adenine, at the base edges of the minor groove floor.^57,58^ AATT-DB1476-I and AATT-DB1476-II show identical interactions with the DNA bases at the minor groove. The BI-N-H in both complexes forms a strong direct H-bond in a bifurcated manner^58^ with the O2s of T7 and T19, respectively (Figure 2). In addition to the strong BI-N-H hydrogen bonds, one of the amidines in each complex, A-N6 in AATT-DB1476-I and C-N4 in AATT-DN1476-II, forms a strong direct hydrogen bond with O2 of C9 (Figure 2). Indirect, water-mediated hydrogen bonding has previously been reported in the diamidine binding of the minor groove of AT sequences.^27,28^ While many classical small molecules like polyamides bind to the minor groove as dimeric and hairpin binding structures,^59−61^ our structural results show that DB1476 is among the select group of small molecules^28,29,62,63^ with the unique feature of utilizing an interfacial water molecule in its monomeric binding of the minor groove. In the X-ray structure of AATT-DB1476-I (Figure 2a), the C-N4 of the DB1476 (Figure 1) uses an interfacial water molecule to form a hydrogen bond with the O2 of T20 (−NH•••O – H•••O=T). Similarly, in AATT-DB1476-II (Figure 2a), the A-N6 (Figure 2a) forms a hydrogen bond with the O2 of T20 (−NH•••O – H•••O=T) via an interfacial water molecule (Figure S2).

In both structural orientations, BI-NH (or B-N2) of DB1476 forms a specific bifurcated with the DNA minor groove. Another remarkable feature of DB1476 is its interaction with water molecules at both terminal amidine ends. DB1476 displaces the spine of hydration in the minor groove of free DNA, AATT, causing an ensemble of water networks that mediate both ligand binding and considerable noncovalent interactions across the minor groove (Figure S3). These water networks are significant because their interactions do not reduce the binding affinity of DB1476 for the minor groove, and more importantly, the arrangement of these waters has been shown to specifically favor AT-rich *B*-form DNAs.^27,28,63,64^ The 11 conserved water clusters previously reported for a bound-AATT minor groove^27^ were also observed in both AATT-DB1476 complexes. The water molecules at the amidine terminal end stabilize the complex via a network of hydrogen bonds that extend across the DNA minor groove from the backbone phosphates to the bases at the floor of the minor groove (Figure S3). The special design characteristics of DB1476 give the compound a very high selectivity for AT base pairs and key functional features that allow for favorable molecular interactions.

### 3.2 MD Simulation Studies of DB1476 Binding Orientation and Interactions in the Minor Groove of −AATT– DNA

To extend the observations of the ligand-DNA X-ray structures, we have carried out 600 ns MD simulation studies to understand the dynamic behaviors of the benzimidazole diamidine and −AATT– sequence complexes (Figure 1). This simulation study provides some new and interesting views of interfacial and terminal bound waters essential for strong DNA complex formation and the local dynamics of minor groove binders. The results also emphasize the correlated effects of the compound and minor groove properties in binding affinity and dynamics.

As shown above, the X-ray structure of AATT-DB1476-I with the −AATT– site uses an interfacial water molecule to help connect DB1476 to the minor groove. MD analysis of this complex also captures the interfacial water and additional compound-water-DNA interactions. In the AATT-DB1476-I complex, the amidine group (N5/N6) at Ph-amidine (A) forms a strong direct H-bond with O2 of C21 (C21=O---N5/N6, 2.8 Å) for more than 91% of the time (Figure 3a) and for the remainder of the time, N5/N6 amidine uses interfacial water to connect with the C21 base (Figure 3b). For the MD analysis, bond lengths from 2.6 to 3.4 Å are associated with direct H-bonding between an amidine and DNA, such as N5/N6 and O2 of C21 (Figure 3b, purple and orange lines). Bond distances of 3.6 to 5.8 Å are the amidine to DNA distances when no direct hydrogen bonding is possible. In this case, an interfacial water-mediated H-bond to the amidine and to the DNA link the compound to the DNA (Figure 3b).

The N3/N4 amidine needs interfacial water for approximately 70% of the time to connect with O2 of T8 (T8 = O---H-O----N4/N3, with average bond distances 2.9, 3.0 Å, respectively, Figure 3a,d). The water molecules in the DB1476 binding site can adequately orient to provide favorable curvature to the DNA complex and interactions between the compound and DNA (Figure 3a). The H-bonding ability and dynamics of the bound waters help provide the high binding affinity of DB1476 to the −AATT– site (1 nM *K*_D_). Due to the bond and angle flexibility of amidine groups, N5/N6 can rotate or flip 180° one time in the 600 ns simulations (Figure 3a,b).

As observed in the crystal structure (Figure 2a), the BI-N2 forms strong bifurcated H-bonds between the O2 of T19 and T7 (Figure 3a,b), and this interaction is very consistent and stable throughout the MD simulations (Figure 3c). The lower oscillation of the BI-N2---O2s (T19 and T7) bond suggests that the central benzimidazole ring is less flexible and stacked on the minor groove tightly.

The complex is also stabilized by the phenyl-CHs of DB1476, which can form stabilizing interactions with O2s of T in the minor groove (Figure 3a). Both amidines are also connected with very dynamic, external, extended terminal water networks in the groove, which further stabilize the close contacts of DB1476 with DNA bases and the sugar-phosphate backbone (Figure 3a).

Based on the 1.63 Å resolution X-ray crystal structure of the AATT-DB1476-II (Figure 2b), we also investigated the MD simulation of DB1476 with the 180° flip orientation (Figures S4 and S5). The MD simulation suggests that the two DB1476 orientations yield similar H-bonding interactions and hydration interactions (Figure 3), as observed in the X-ray structures (Figure 2).

### 3.3 X-ray Complex Structure of AAATTT-DB1476 and Its Minor Groove Interactions

**Figure 4:**
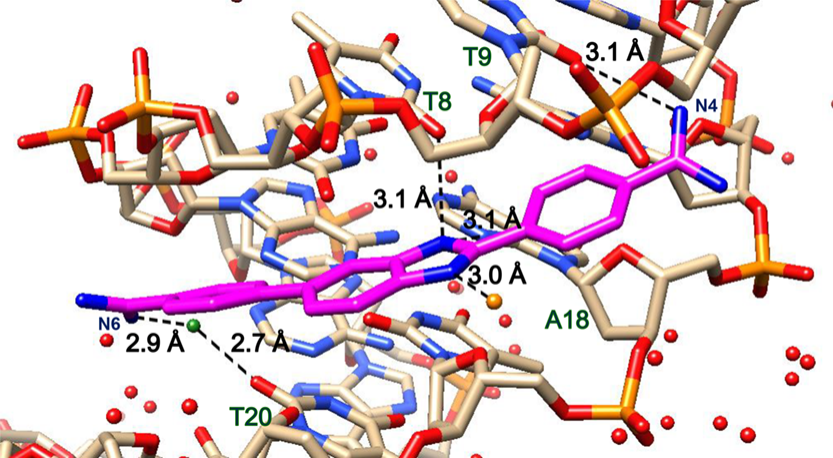
Significant bonding distances in the structure of **AAATTT-DB1476**. DNA in tan and DB1476 in magenta. A bifurcated hydrogen bonding between BI-N2 (blue) with O2 (red) of T8 and N3 of A18 with 3.1 and 3.1 Å bonding distance, respectively. The direct hydrogen bonding distance between C-N4 and O2 of T9 is 3.1 Å. The interfacial water-mediated (green, black lines) bonding distance from A-N6---O-H_2_ is 2.9 Å and from O-H_2_---T20 is 2.7 Å. The strong hydrogen bond between the “unique external water” molecule (in orange) and B-N1 is 3.0 Å.

The AAATTT-DB1476 complex structure is reported at 1.54 Å with *R*_work_ and *R*_free_ at 20.4 and 24.2%, respectively. In the AAATTT-DB1476 structure (Figures 4 and S6), the central B-N2 forms a strong direct hydrogen in a bifurcated manner with the O2 of T8 and the N3 of A18, respectively (Figure 4). The C-N4 of the ligand (Figure 2) forms a strong direct hydrogen bond with the O2 of T9 (Figure 4). The A-N6 forms an H-bond with the O2 of T20 via an interfacial water-mediated contact. The X-ray structures show that DB1476 binding in AATT and AAATTT spans across five residues in the minor groove, emphasizing the unique selectivity and structural property of DB1476. Like the former complex, the AAATTT-DB1476 structure presents a surrounding network of water molecules from the backbone phosphates (T19) across the C-N4 terminal end to the base edges of the minor groove (Figure 4). Terminal water molecules are also observed at the A-N6 amidine end. Notwithstanding the similarities, our structural results did reveal a major difference between the AATT-DB1476 and AAATTT-DB1476. We found a ″unique external water″ molecule (*orange* in Figure 4), previously not observed in −AATT– that forms a very strong direct H-bond (3.0 Å) with B-N1 of the benzimidazole. The finding of this external water molecule is crucial for two reasons: (i) In the AATT-DB1476 complex structure, no other water molecules (at 1σ of intensity) with a favorable hydrogen bonding distance were observed in proximity to B-N1 and B-N2 of the benzimidazole and (ii) the nonhetero ring of the benzimidazole is linked to another phenyl ring which makes a surrounding water molecule less probable. Therefore, any water molecule with an H-bond capacity with B-N1 plays a critical role in stabilizing the ligand (Figure S7). Thus, the spatial location of this “unique external water” molecule and its strong hydrogen bond with B-N1 locks in the orientation of the DB1476. No other interaction, except for B-N1, can hold the external water in that location. The reverse orientation (180° flip) of DB1476 shows that the external water molecule has no available hydrogen bonding interaction needed to stabilize the AAATT-DB1476 complex structure. More so, the reverse DB1476 orientation does not have an optimum structural fit of its electron density. Hence, our results confirm only one possible (or favorable) orientation for the AAATTT-DB1476 complex structure. These results suggest that the AAATTT minor groove provides a more favorable binding interaction than AATT for DB1476. Biosensor-SPR results (Table S4) show that AAATTT binds DB1476 twice (*K*_D_ = 0.5 nM) as strongly as AATT.

### 3.4 X-ray Structures of Alternating AT DNAs and Their Minor Groove Interactions

**Figure 5:**
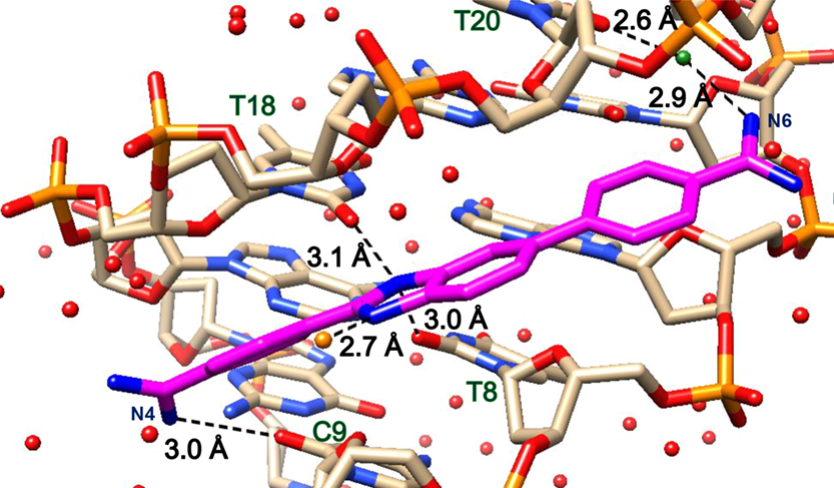
Significant bonding distances in the structure of **ATAT-DB1476**. DNA in tan and DB1476 in magenta. A bifurcated hydrogen bonding between benzimidazole N2 (blue) with O2 (red) of T8 and T18 is 3.1 Å and 3.0 Å, respectively. The direct hydrogen bond between C-N4 and O2 of C9 is 3.0 Å. The interfacial water-mediated (green, black lines) bonding from phenyl-amidine A-N6---O-H_2_ is 2.9 Å and from O-H_2_---T20 is 2.6 Å. The hydrogen bond between “external water” molecule (in orange) and B-N1 is 2.7 Å.

DNA solution studies,^65−68^ and structure determination,^65−68^ have all reported a wider minor groove for the alternating AT sequences in comparison to narrow AT A-tract sequences.^69−71^ Interestingly, the structure determination of the native dodecamer d(5′-CGCGATATCGCG-3′)_2_ has been unsuccessful. Here, we report a novel alternating AT native d(5′-CGCGATATCGCG-3′)_2_ structure (Figure S8) with a narrow minor groove at a resolution of 1.88 Å. We also describe the high-resolution X-ray structure of ATAT-DB1476 at 1.63 Å, with *R*_work_ and *R*_free_ at 18.3 and 20.1%, respectively. Like AAATTT-DB1476, the 1:1 complex structure of ATAT-DB1476 shows one favored orientation of the DB1476 model (Figure 5) with strong direct hydrogen bonds at the −ATAT– minor groove (Figures 5 and S9). The specific and direct strong bifurcated hydrogen bonding between the B-N2 and O2 of T18 and T8, and the strong H-bond between C-N4 an O2 of C9 (3.0 Å) marked the DB1476 a strong ATAT (*K*_D_ = 12 nM) (Table S4) binder. The bifurcated hydrogen bonding in the ATAT-DB1476 X-ray structure differs from the AATT-DB1476 structure in that N-H of the BI binds slightly further down the DNA (T7 in −AATT–, T8 in −ATAT−). ATAT-DB1476 shows an “external water” molecule. The unique external water stabilizes the ATAT-DB1476 complex structure by forming an H-bond with B-N1 (Figure 5), confirming the singular DB1476 orientation at the −ATAT– minor groove (Figure S10). Due to the moderate flexibility and crescent-shaped DB1476, the direct strong H-bond at one amidine end (C-N4) allows the other end (A-N6) to form an indirect water-mediated H-bond with O2 of T20. This binding pattern of DB1476 is consistent with the already described nonalternating AT complex structures (Figures 2 and 4). The terminal ends of the amidines have a surrounding water network that stabilizes the DNA-ligand interactions (Figure 5).^27^

**Figure 6:**
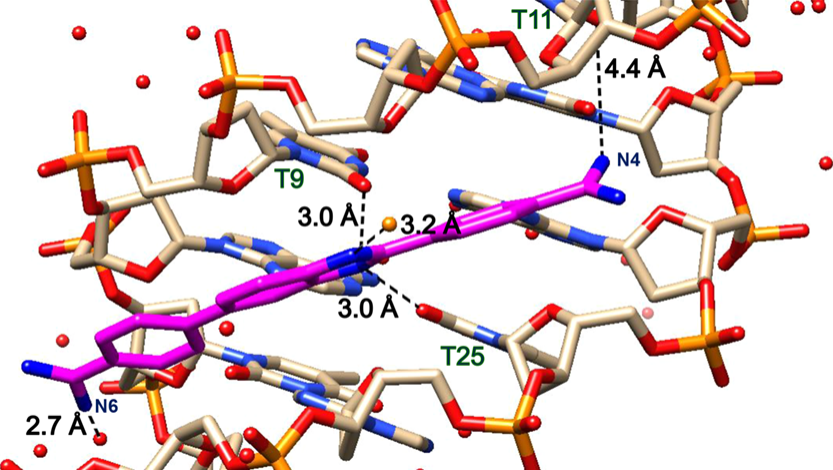
Significant bonding distances in the structure of **ATATAT-DB1476**. DNA in tan and DB1476 in magenta. The bifurcated hydrogen bonding between BI-NH and O2 (red) of T9 and T25 is of 3.0 Å and 3.0 Å, respectively. Hydrogen bonding between external water and B-N1 (blue) is 3.2 Å.

An extended alternate AT sequence (ATATAT) is also described. The previously reported ATATAT native structures have not been determined at a high resolution. Here, we report a 16-mer ATATAT-DB1476 complex structure at a resolution of 1.50 Å, with *R*_work_ and *R*_free_ at 25.9 and 29.2%, respectively (Figures 6, and S11). The crystal structure has the space group P3_1_21, which is different from all other structures in this study. The DB1476 compound binds in a singular orientation, like AAATTT and ATAT, in the presence of a unique external water molecule that stabilizes the complex at the B-N1. A bifurcated hydrogen bond is observed between the BI-NH and the O2 of T9 and T25, respectively (Figure 6). The C-N4 amidine has a longer than expected bonding distance from their nearest DNA hydrogen bond acceptor. The interfacial water molecule observed at A-N6 does not have an optimum H-bond distance from the DNA. The unusual bonding distances suggest a very dynamic interaction at the amidine ends, and therefore, DB1476 will not bind as strongly to the −ATATAT– (*K*_D_ = 16 nM) (Table S4) sequences in comparison to −ATAT– (*K*_D_ = 12 nM) (Table S4). The ATATAT-DB1476 structure utilizes its extensive large water network to stabilize its complex.

### 3.5 X-ray Structures of Pure A-Tract Complexes and Their Minor Groove Interactions

**Figure 7:**
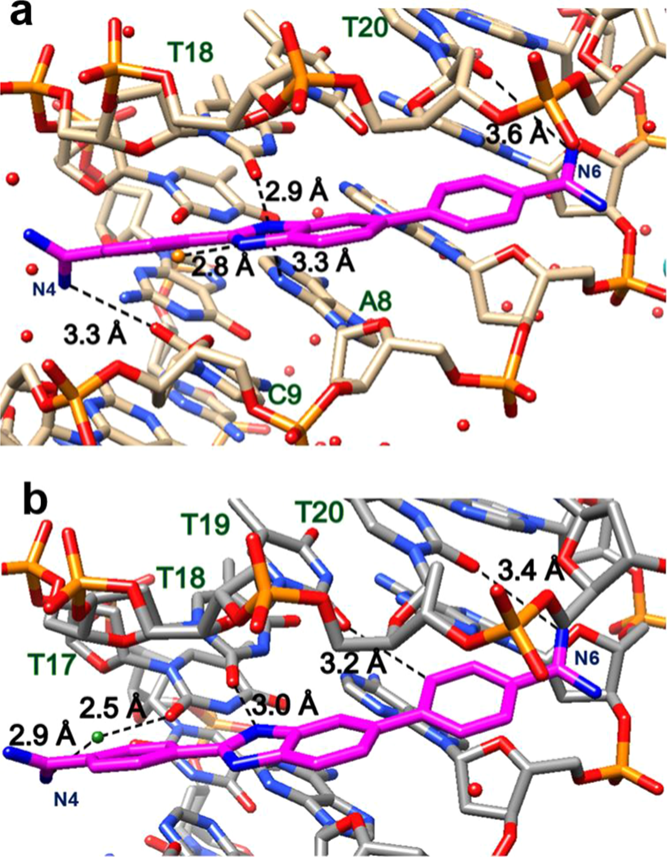
(a) Significant bonding distances in the structure of **A4-DB1476**. DNA in tan and DB1476 in magenta. A bifurcated hydrogen bonding between B-N2 (blue) and O2 (red) of T18 and N3 of A8 is 2.9 Å and 3.3 Å, respectively. A strong directed bond between C-N4 and C9 is 3.3 Å. The hydrogen bonding between external water and B-N1 is 2.8 Å. The bonding distance between A-N6 and O2 of T20 is 3.6 Å. (b) Significant bonding distances in the structure of **A6-DB1476-II**. DNA in gray and DB1476 in magenta. A strong hydrogen bond of 3.0 Å is observed between B-N2 (blue) and O2 (red) of T18. The interfacial water-mediated (green, black lines) bonding from phenyl-amidine C-N4---O-H_2_ is 2.9 Å and from O-H_2_---T17 is 2.5 Å. A-N6 contacts O2 of T20 at 3.4 Å. Favorable contact between B-C19 and O2 of T19 (3.2 Å).

For simplification, the −AAAA– and −AAAAAA– DNA sequences are identified as the A4 and A6 DNA sequences hereafter respectively. The A4-DB1476 complex structure is reported at 1.55 Å, with *R*_work_ and *R*_free_ at 22.0 and 24.7%, respectively (Figure S12). The minor groove binder, DB1476, binds to the asymmetric A4-DNA sequence in a singular orientation. The presence of stabilizing external water, like in the −A3T3– and −ATAT– sequences, necessitates the selection of a favored orientation of DB1476. The described prominent features of DB1476 like the BI-N-H bifurcated hydrogen bonding and the strong direct hydrogen at one amidine (C-N4) remain consistent in the binding of the A4-DNA minor groove (Figure 7a). The amidine A-N6 has a bonding distance of 3.6 Å from the DNA, suggesting the presence of water-assisted hydrogen bonding, which is expected from the curvature of the ligand. However, from the structure, the waters in the region appear too dynamic to be observed from the X-ray structure. Nonetheless, the presence of an extensive water network across the minor groove stabilizes the A4 complex relatively well (*K*_D_ = 15 nM) (Table S4).

The A6-DB1476 complex is reported at a resolution of 2.1 Å (Figures 7b and S13). The electron density map showed no clear preference for one DB1476 orientation. Therefore, the ligand was refined in two orientations within the ligand’s electron density map, and both orientations fit the density somewhat similarly (Figure S14). The final statistics show that A6-DB1476-I and A6-DB1476-II have slightly different *R*_free_ values, 31.0 and 32.5%, respectively, and different average B-factor values, 48.8 and 45.1, respectively. The B-factor being higher than usual is most likely from the noncomplementarity of the A6 sequence (Figure S14).

Since the structures of both complexes are very similar, only A6-DB1476-II will be described subsequently. The B-N2 of A6-DB1476-II forms a strong hydrogen bond with the O2 of T18 (3.0 Å) (Figures 7b and S13). The amidine A-N6 forms a hydrogen bond with the O2 of T20 (3.4 Å). The bond length is slightly longer than the acceptable limit. The C-N4 amidine forms a hydrogen bond with the O2 of T17 of the DNA via an interfacial water molecule (Figure 7b). Because of the low resolution (2.1 Å) of the A6-DB1476 complex, only 10 water molecule peaks (including a water molecule at the C-N4 terminal end) were observed within one sigma of the 2*F*_o_–*F*_c_ map. Notwithstanding, DB1476, again, shows a remarkable selectivity for another variety (A6 sequence) of AT-rich DNA. Other pertinent interactions in the A-tract minor groove include the benzimidazole’s phenyl-CHs making good contact with the O2 of T19 and the DNA backbone (Figure 7b).

### 3.6 X-ray Complex Structure Comparisons of a DB1476 Indole Analogue, DB1884

**Figure 8:**
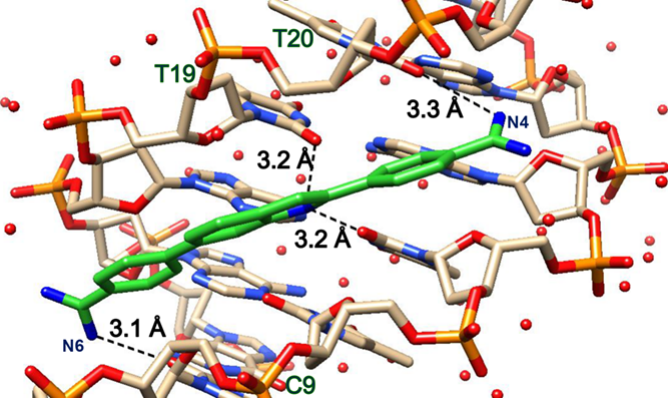
Significant bonding distances in the structure of **AATT-DB1884**. DNA in tan and DB1884 in green. The bifurcated hydrogen bonding distances between benzimidazole, N2 (blue) with O2 (red) of T7 and T19 are 3.2 and 3.2 Å, respectively. The direct hydrogen bond between A-N6 and O2 of C9 is 3.0 Å. Interfacial water not observed between C-N4 and O2 of T20. Direct contact made by C-N4 with O2 of T20 with 3.3 Å H-bond distance.

**Figure 9:**
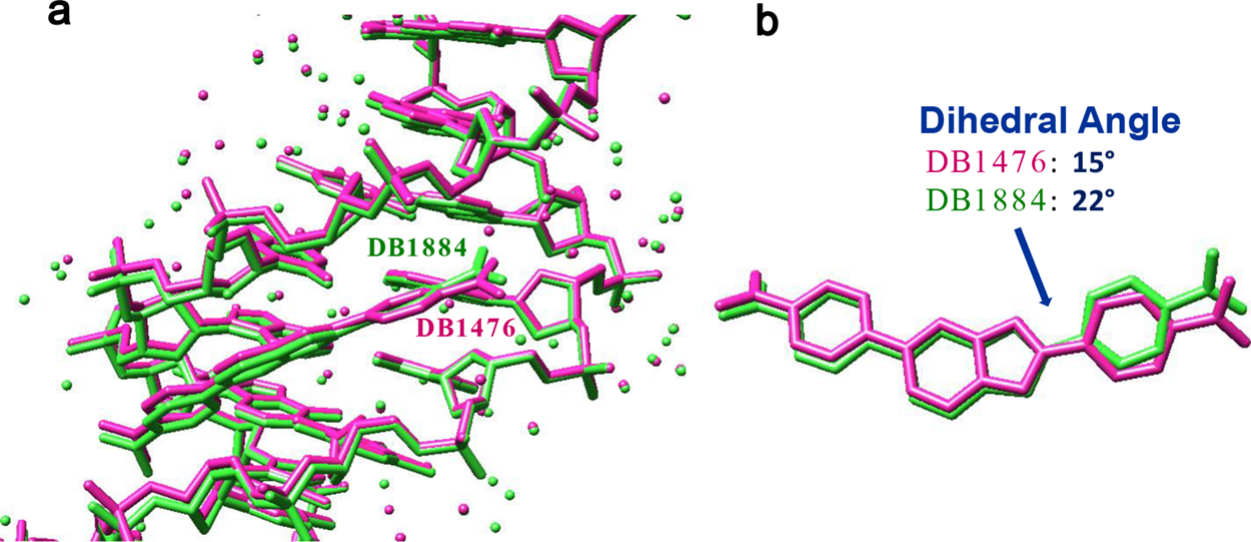
(a) Overlay of DB1476-I and AATT-DB1884 in the minor groove. AATT-DB1476 in magenta, and AATT-DB1884 in green (b) Overlay of DB1476 and DB1884 without global DNA structure.

To further evaluate the unique characteristics of DB1476, its minor groove interactions are compared with a similarly strong AT binder, an Indole-diphenyl diamidine, DB1884. The X-ray structure of AATT-DB1884 (Figure 8) and crystallographic statistics (Table S3) shows that DB1884 behaves closely like DB1476 in the AATT minor groove. DB1884 binds to the minor groove of AATT in the same orientation as AATT-DB1476-I (Figure 9a). The BI-N2 of the indole forms a bifurcated hydrogen bond with T7 and T19, respectively. The A-N5 forms a strong hydrogen bond with the O2 of C9 at 3.1 Å (Figure 8). The distance between the C-N3 of the ligand and O2 of T20 is 3.3 A. When comparing the complex structures of AATT-DB1884 and AATT-DB1476, a significant difference in their minor groove-binding is observed (Figure S15). DB1476 requires an interfacial water to form complexes with the pure AT DNAs, but DB1884 is attached to the DNA bases without the assistance of an interfacial water (Figure 8). The reason for the DB1476 preference for an interfacial water-assisted binding will be addressed in the discussions.

### 3.7 Analysis of DNA Structural Parameters

AT-rich DNA sequences correspond to the basic *B*-form model derived from fiber diffraction experiments^72,73^ but with significant variation in the local minor groove structure. The X-ray structures and characteristics of *B*-form DNA have demonstrated that the local structural conformation of DNA is dictated by its base pair sequence.^74^ Many of the reported structures of AT sequences generally have a narrower minor groove^75^ with some results showing that an increase in the propeller twists of AT sequences can lead to a narrower minor groove.^75−77^

**Figure 10:**
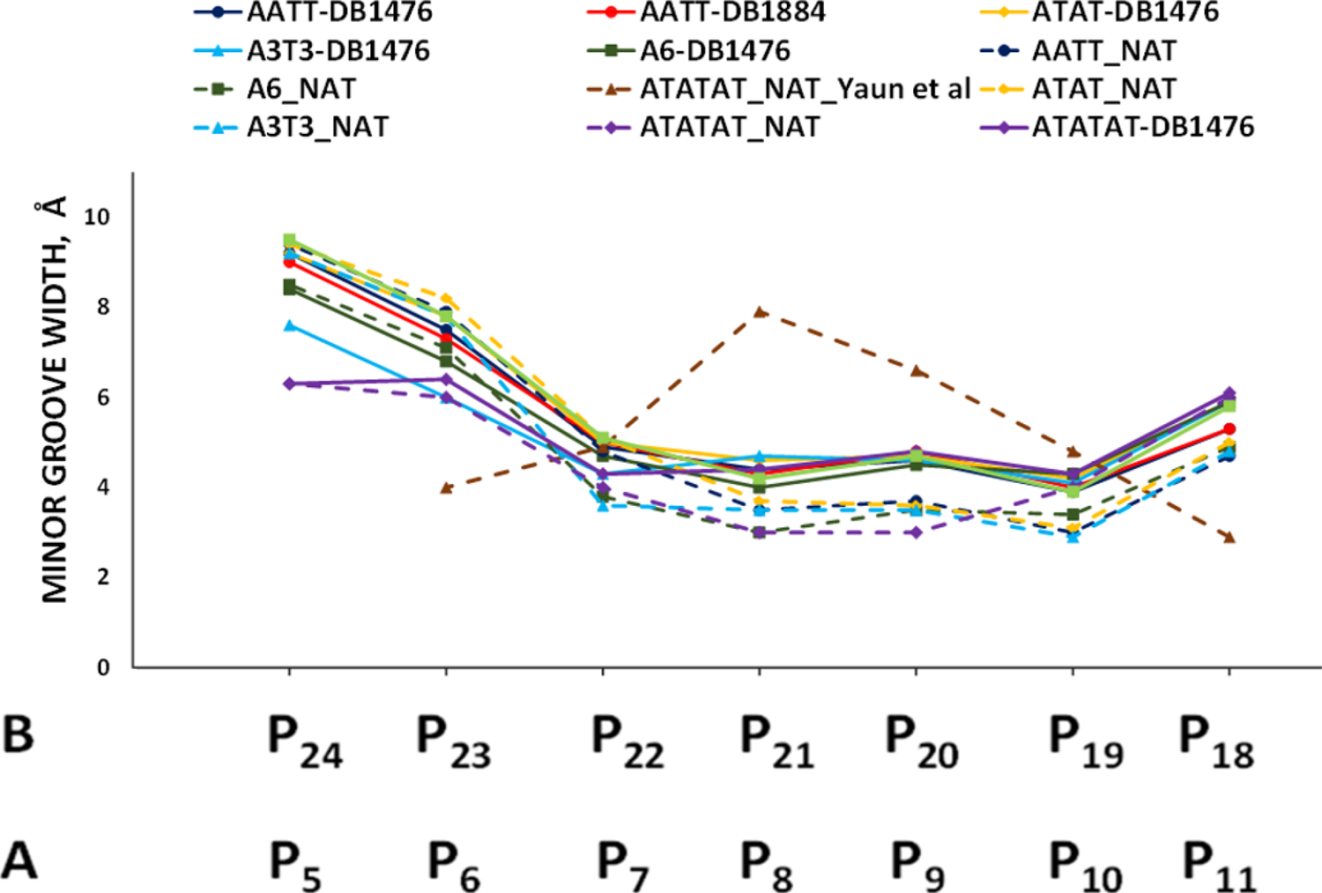
Minor groove distance comparison of the reported DNA-ligand. The presence of ligand in the complexes widens their respective minor grooves along interacting bases. AT DNA-DB1476 complex in solid line, native in a dashed line. ATATAT_10mer native structure (Yuan et al.,^69^).

The results from Figure 10 show a widening of the minor groove of AATT-DB1476, AATT-DB1884, AAATTT-DB1476, ATAT-DB1476, ATATAT-DB1476, A4-DB1476, and A6-DB147 complexes compared to their native DNA structures. Although the ATAT native DNA shows a narrow groove width, it still has a wider minor groove relative to the rest of the sequences (Figure 10). Previously reported solution studies do support a wider minor groove for native alternating AT sequences.^67,68^ In addition, Yuan et al., report a native ATATAT decamer (10-mer) X-ray structure with a very wide minor groove.^69^ In contrast, Yoon et al., reported a native ATATAT dodecamer (12-mer) with a narrow minor groove width.^77^ The reason for the groove differences between the 10-mer and 12-mer ATATAT structures is suggested to arise from the different motif packing arrangements within the crystal lattice.^77^ Our experiments to additionally investigate the minor groove of alternating AT DNA sequences using d(5′-GCTGGATATATCCAGC-3′)_2_ resulted in a structure with a narrow minor groove width similar to that of the dodecameric structure (Figure 7). In this report, we show the two native alternating AT DNAs,16-mer ATATAT and 12-mer ATAT, to have a narrow minor groove (Figure 10). However, the 12-mer ATAT DNA is wider. Overall, the many reports regarding alternating AT sequences lead us to conclude that ATATAT DNA crystal structures can have both wide and narrow minor grooves depending on the experimental crystallization conditions and local packing interactions. Crystallization of DNA molecules selects energetically similar DNA molecules that can pack in an efficient periodic manner to form a crystal structure. DB1476 binds ATAT and ATATAT to form a complex that correlates with the minor groove of the AT-tract DNA (AATT, AAATTT, A4, A6) complexes. These preliminary results suggest that DB1476 binds similarly to most AT sequences (Figure 10).

**Figure 11:**
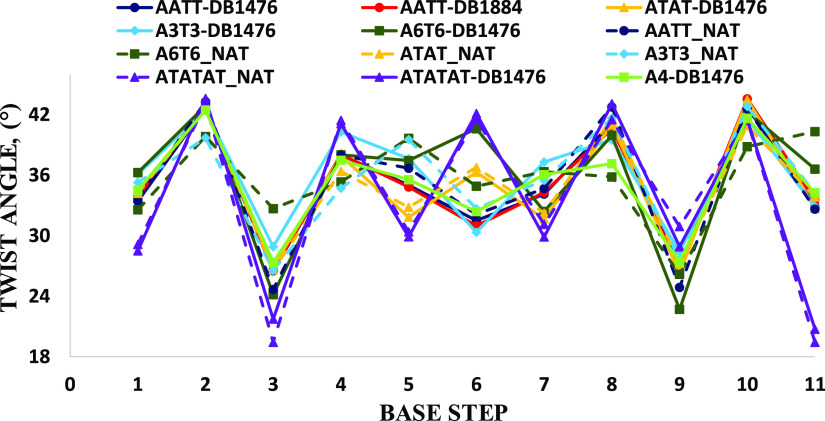
Plot of base step helical twist for all the studied DNA-DB1476 complexes. AT DNA-DB1476 complex in solid line, native in dashed line. A6-DB1476 (solid green) the highest twist and is significantly different from native structure (dash green). ATAT-DB1476 (solid orange) shows an alternating twist in the minor groove, native ATAT is similar (dash orange). Apart from A6-DB1476 complex, there is no significant difference between DB1476-bound DNA and native structures.

Base step parameters like the helical twist, roll, and slide are important structural properties to consider when analyzing the general structure of DNA.^76−79^Figure 11 shows a modest difference in DNA twist in the AT regions of ATAT-DB1476, ATATAT-DB1476, and A6-DB1476 compared to the other complex structures. These other structures from Figure 11 do not show any appreciable changes in the twist in their AT regions following the binding of DB1476. Only the native A6-DNA shows a considerably lower twist than its complex at steps 6 and 8 (Figure 11).

**Figure 12:**
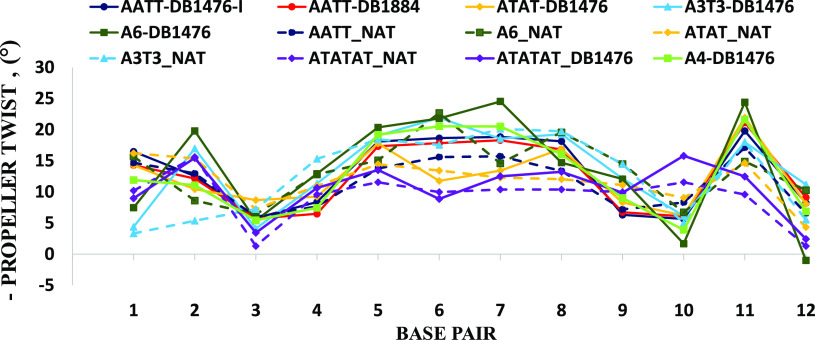
Plot of base pair propeller twist for all the studied DNA-DB1476 complexes. AT DNA-DB1476 complex in solid line, native in a dashed line. A6-DB1476 (solid green) the highest propeller twist and is different from native structure (dash green) except at step A6. Apart from A6-DB1476 complex, there is no significant difference between DB1476-bound DNA and native structures.

The propeller twist, an important base pair parameter, was calculated for all the structures to determine the twist of the base pairs across the minor groove following DB1476 binding. The propeller twist values of AATT-DB1476, A3T3-DB1476, ATAT-DB1476, ATATAT-DB1476, A4-DB1476, and A6-DB1476 complexes do show differences in the pure AT region (Figure 12). However, like the twist parameter, the native structures show no major differences in their propeller twists in comparison to their complexes (Figure 12). More base pair parameters like the DNA roll also show no significant changes between the native DNA and their bound forms (Figure S16).

Although the binding of DB1476 on AT sequences widens the minor groove of the global native DNA structure, DB1476 does not exert any significant changes on their local structural properties. These results suggest that the structural parameter differences among the various DNA-DB1476 complexes are entirely sequence-dependent.

## 4 Discussion

X-ray crystal structure results show the binding of DB1476 to the minor groove of −AATT– is possible in two orientations (N6-BI-N4 or N4-BI-N6) along the C1:G12 direction (Figure 2, Figure S3). The 2*F*_o_–*F*_c_ map of DB1476 shows an excellent occupancy at one sigma (Figure S1). The rational and effective design of DB1476 makes for an efficiently sized, moderately flexible, and functionally specific compound. The MD results (Figure 3) show that the shape, flexibility, and cationic charge of DB1476 allow it to sufficiently re-orient and adopt favorable positions across the minor groove. The various hydrogen bonding interactions, both direct and water-mediated, between DB1476 and the DNA at the many dynamic states of the MD simulation, (Figure 3) confirm the two possible orientations for DB1476 binding in the minor groove. Both structural and MD results show that the BI-N-H hydrogen bond formation with O2s of T19 and T7 determines the recognition code of DB1476. The B-N2 hydrogen bond is not only strong (Figure 2) but is remarkably stable for the entire MD simulation (Figure 3b). Furthermore, results show that the formation of a strong, direct, and stable H-bond at one amidine terminal (−N···O=T), induces a conformation of DB1476 that makes a strong direct H-bond at other amidine terminal less probable, and the use of an interfacial water molecule is very necessary for DNA contact (Figure S1a). The dynamics of DB1476 cause the compound to adopt a conformation where one amidine end becomes much closer to the minor groove floor. The case is also the same in a different orientation (Figure S1b).

An unusual feature of the −AATT– complex is that DB1476 slides one bp out of the AATT sequence to form a strong direct H-bond to the O2 of C9 in the C9-G16 bp. The reason for the GC interaction must have much to do with the interaction of DB1476 with the groove shape at the end of the AATT sequence. The wider groove at this position favors DB1476 binding. At the other end of the complex, DB1476 forms a water-mediated H-bond to O2 of T20 in the T20-A5 bp. This water-mediated H-bond may also be partly responsible for the GC bp recognition at the other end of DB1476.

In the MD analysis of the DB1476-AATT site, the end at the GC bp is also found to form a strong direct C=O H-bond over 90% of the time, confirming our crystal structural results. The other end of the complex is primarily water-mediated, as seen in the X-ray structure. The 180° rotated ligand behaves in a remarkably comparable manner as with the former X-ray results.

The structure of the AAATTT-DB1476 complex shows the binding of DB1476 in one orientation (Figure 4). Small AT-specific molecules like distamycin and Hoechst dye have shown similar interactions with −AAATTT–.^80,81^ The DB1476 orientation of N6-BI-N4 along the C1:G12 end is stabilized in its orientation by an external water molecule bound to B-N1 and thus affirms the preferred structural orientation of DB1476. This N6-BI-N4 orientation of DB1476 along the C1:G12 direction favors a strong direct bond H-bond of C-N4 with T9, and an interfacial water-mediated H-bond of A-N6 with T20 (Figure 4). Like most AT-tract minor grooves, AAATTT DNA has a high propeller twist in the AT region, which in many cases correlates to a narrower minor groove. The high-resolution X-ray structure from Williams’ group also confirms that longer AT-tract (AAATTT) leads to a narrower minor groove whose shape has a greater affinity for planar heterocyclic compounds.^82^ As a result, the strong binding of DB1476 is favored (*K*_D_ = 0.5 nM).

The initial netropsin structure with d(CGCGATATCGCG)_2_, has many of the same interactive features as with −AATT– but also some significant differences. In the structural refinement of the −ATAT– complex the fit of netropsin to the electron density required two opposite orientations in the −ATAT– site. The two −ATAT– structures had broadly similar interactions with the DNA sites. The two orientations agree with an NMR solution analysis of the netropsin complex with −ATATAT– that clearly shows a rapid exchange between equivalent binding sites for netropsin that does not involve global dissociation from the DNA binding site.^83^ Surprisingly, netropsin could flip 180° to provide the two orientations of the bound compound without complete dissociation from the DNA site. Flipping of the compound agrees with the two orientations observed in our AATT-DB1476 X-ray results. Another result with the −ATAT– complex casts some doubt on the requirement for a high propeller twist to give a narrow minor groove. Two of the four AT base pairs in the ATAT stretch, for example, have low propeller twist angles, even though the DNA has a narrow minor groove. Since there was no previously reported structure of the −ATAT– free DNA in the literature, it is difficult to compare the change of groove width in the ATAT-netropsin complex. As noted earlier, studies suggest that the minor groove width in the free −ATAT– site is significantly wider than observed in −AATT–. Calculation of the groove widths of the d(5′-CGCGAATTCGCG-3′)_2_ and d(5′-CGCGATATCGCG-3′)_2_ indicate a significantly wider minor groove in −ATAT– than in −AATT–.^25,26^

However, in our studies we have observed that the X-ray structure of the ATAT-DB1476 complex has a narrow minor groove (Figure 5), and the BI-N-H can form a bifurcated hydrogen bond with T8 and T18 (Figure 5). The functionality at the DB1476 terminal allows direct hydrogen bonding to C9 and water-mediated hydrogen bonding to T20. Biosensor-SPR binding studies show that DB1476 binds ATAT about 10 times weaker than AATT (Table S4). Yet DB1476 binding to the ATAT site is favored in one orientation in contrast to the AATT DNA. The selection of a particular orientation is not entirely understood, but we observe that the repeating symmetry units across the P2_1_2_1_2_1_ crystal lattice require an N6-BI-N4 orientation of DB1476 along the C1:G12 direction. Also, the phenomena of crystal packing may help explain why our alternating AT DNAs have a narrower minor groove in X-ray structures but give a wider minor groove from solution structures. Yuan et al. cite crystal packing effects as the reason for the wider minor groove in the native 10-mer ATATAT structure. Crystals usually require stabilized molecules in an optimum condition for growth. Therefore, the process of crystal growth can be reckoned as a “selection process” for energetically stable DNA molecules. In solution, the ATATAT DNA is more dynamic, with duplexes that can have wider minor grooves. Hence, the reported solution structures may not be thermodynamically stable with a wider minor groove. In contrast, the X-ray structures of ATAT-DB1476 and ATATAT-DB1476 have narrower minor grooves. The hydrogen bonding, electrostatic interactions, and van der Waals forces between DB1476 and the minor groove confer significant stability to the complex. Both binding and structural results confirm that the DB1476 compound binds ATAT better than ATATAT (Table S4).

The A4-DB1476 complex structure shows similarity with the AATT-DB1476-II structure in that DB1476 binding extends outside the AT-tract (C-N4 bond with C9, Figure 7a). A bifurcated hydrogen bonding, a strong direct H-bond, and an amidine terminal water network stabilize both structures. This suggests the asymmetric A4 DNA, compared to the symmetric AATT, has not caused any notable change in the AT recognition by DB1476 and the overall DNA global structure.

Nonetheless, with more structural data, a crucial trend begins to emerge. The A4-DB1476 structure reveals the DB1476 binds in the N6-BI-N4 orientation of DB1476 along the C1:G12 direction. Therefore, for all the complex structures with a fixed orientation of DB1476, a strong direct bonding of the amidine to the DNA seems to occur at C-N4, suggesting that the amidine C-N4 is favored to bind directly to the DNA while C-N6 will utilize interfacial water for DNA contact. This unique binding behavior of DB1476 is now observed in nonalternating, alternating, and pure A-tract AT DNA sequences. Although the reason for this preferential binding is not yet understood, one possibility could be the proximity of BI-N1 and BI-N2 of the benzimidazole to the C-N4 amidine. The increase in molecular interactions, from hydrogen bonding and electrostatic forces, around the heterocyclic ring of the benzimidazole will lead to an uneven distribution of charge in that region, increasing its affinity for the electronegative atoms in the minor groove.

The A6-DB1476-II complex still shows a consistency in the AT recognition pattern of DB1476 across AT sequences. The binding of DB1476 spans across five successive adenine residues beginning from adenine 5 (−AAAAAG−). The BI-NH forms a strong hydrogen bond (3.0 Å) with the O2 of T18 and uses a water molecule for an H-bond with the O2 of T17 (Figure 7b). However, the A-tract complex does show a significant discrepancy. The abundance of O2 of thymine protruding from the floor of the minor groove seems to direct the hydrogen bond interaction between the DB1476 and DNA. So far, for all AT DNA-ligand complexes in this study, both amidine terminals of DB1476 form H-bonds with the helix strand A and helix strand B, respectively. However, in both A6-DB1476 structures, the two amidines in each of both DB1476 orientations participate in an H-bond interaction with only strand B. The DB1476 preference for one strand is not completely understood since the compound can readily form strong hydrogen bond with the available N3 of adenine in the minor groove. The MD studies of AATT-DB1476 and structural results from all other AT complexes have shown that the predominant pattern of DB1476 binding involves an optimum H-bond interaction of both amidines with strand A and strand B, respectively. These comprehensive results confirm that the unique orientation of DB1476 in the minor groove is necessary for its binding and overall DNA complex stability. Therefore, as much as DB1476 may have a preferential binding orientation across the minor groove, the structure of A6-DB1476 suggests that DB1476 can still adopt differential binding configurations in the minor groove of different AT DNA sequences to account for unfavorable interactions. The dynamics of DB1476 in the A-tract minor groove may explain why the H-bond distance between A-N6 and T20 is 3.4 Å.

The AATT-DB1884 complex structure shows similarity with the AATT-DB1476, with the major difference being the water-assisted binding of DB1476 to its DNA (Figure 8). The reason for this difference lies in the greater curvature of DB1884 compared to DB1476, which is due to the difference in the bond angles between their five-membered ring (imidazole for DB1476, pyrrole for DB1884) and the attached phenyl group, respectively (Figure 9a). Both ab initio calculations and analysis of the X-ray structures of the two compounds bound to the −AATT– site show an increased bond angle in the indole compared to the benzimidazole (Figure 9b). The indole −CH– relative to the benzimidazole −N– increases the bond angle slightly, an increase of about 7°. This increased angle and molecular curvature places the amidine on the DB1884 phenyl closer to the floor of the minor groove and decreases the requirement of an interfacial water-assisted H-bond (Figure 9).

### 4.1 Structural Parameters

The results show that DB1476 binding of AT sequences alters the DNA groove structure (Figure 10). Since the alternating ATAT and ATATAT native DNA structures favor a narrow minor groove in their crystal packing arrangement, the binding of DB1476 in both the alternating and A-tract AT resembles a classical *B*-form complex (10 bp per turn). Therefore, the minor grooves of −AATT–, −AAATTT–, ATAT, ATATAT, −A4–, and −A6– complexes were all widened by an average of about 1 Å. The noncovalent interactions and minor groove width results confirm that DB1476 can recognize a variety of AT-rich sequences in a similar way.

All the A-tract sequences except for A6-DB1476 have a similar average DNA twist of 31° in their AT region. A6-DB1476 has a higher DNA twist in the minor groove, suggesting an overall increase in rigidity. The A6 DNA rigidity has been shown to cause a conformational state that prevents DNA coiling and DNA digestion by enzymes.^84−86^ In contrast, DB1476 binding of A4 DNA does not seem distinguishable from other AT sequences. The results show that increasing the adenine from four to six does affect certain structural parameters, but not others.

The ATAT and ATATAT DNAs show alternating AT DNA twist characteristics. ATAT-DB1476 has a characteristic normal twist at the TpA step (36°) that is flanked on either side by a lower twist from two ApT steps (31°), respectively. ATATAT-DB1476 also shows the same twist pattern with a high twist at the TpA step (42°) that is flanked by a lower twist from the two ApT steps (30°), respectively (Figure 11).

There are no significant differences between the propeller twists of the free and bound DNA sequence. Nonetheless, the AT-tract sequences do have a higher propeller twist than the alternating AT sequences (Figure 12).

Other base pair and base step parameters show no significant changes or disruption in the global structure of their DNAs following DB1476 binding (Figures S16–S18).

In summary, the detailed structural, dynamics, and binding affinity studies of two minor groove binders with six different DNAs at AT binding sites provide better understanding of AT sequences’ effects on AT-specific ligand binding activity. Such information could help in the design of new minor groove binders to selectively recognize different AT DNA sequences. These sequences are common in the human genome^6,7,24^ and form a major part of the DNA in parasitic organisms such as plasmodium and trypanosomes.^87−89^ Based on these results, an entirely new class of minor groove binders can be developed that incorporate a bound water molecule into their DNA minor groove complex and form a very strong ternary complex.

## Supporting Information Available

2*F*_o_–*F*_c_ maps for DB1476 binding in the minor groove of d(5′-CGCGAATTCGCG-3′)_2_,) X-ray structure of AATT-DB1476-I, MD simulations of DB1476-flip-d(CGCGAATTCGCG)_2_, complex, Dihedral Angle plot of DB1476 at −AATT– minor groove, X-ray structure of AAATTT-DB1476, 2*F*_o_–*F*_c_ map for DB1476 binding in the minor groove of d(5′-CGCGAATTCGCG-3′)_2_, X-ray structure of ATAT DNA with surrounding water network, X-ray structure of ATAT-DB1476 with surrounding water network, 2*F*_o_–*F*_c_ map for DB1476 binding in the minor groove of d(5′-CGCGATATCGCG-3′)_2_, X-ray structure of ATATAT-DB1476, X-ray structure of A4-DB1476, X-ray structure, X-ray overlay structures of AATT-DB1476-I and AATT-DB1884 complexes, plot of base step roll for all the studied DNA-DB1476 complexes, Plot of base step rise for all the studied DNA-DB1476 complexes, plot of base step slide for all the studied DNA-DB1476 complexes, table of crystallization set-up conditions, table of crystallization collection source and details, table of X-ray crystallographic information for DNA-ligand complexes, and table of biosensor-SPR equilibrium dissociation constants (*K*_D_, nM) of DB1476 with pure A·T sequences (PDF)

## Notes

Crystallography data are available from the Protein Data Bank (https://www.rcsb.org/) with access codes 8EC1, 8ED6, 8FDQ, 8EDA, 8EDB, 8FDR, 8F1S, 8F1V, 8FB4, 8F2W, 8FDP, 8F2Y, 8F20, 8F94.
