# Supplementary material for: X-ray Structure Characterization of the Selective Recognition of AT Base Pair Sequences
Source: ACS Bio Med Chem Au. 2023 Apr 5;3(4):335–48. doi: 10.1021/acsbiomedchemau.3c00002 (PMC10436263; doi:10.1021/acsbiomedchemau.3c00002)
Supplement: Supplementary file 1 — bg3c00002_si_001.pdf [file bg3c00002_si_001.pdf]

Supporting Information

X-ray Structure Characterization of the Selective Recognition of AT Base Pair Sequences

Edwin N. Ogbonna,[a,†] Ananya Paul,[a,†] Abdelbasset A. Farahat [a,b,c] J. Ross Terrell,[a] Ekaterina Mineva, [a] Victor Ogbonna, [a] David W Boykin,[a] W. David Wilson[a*]

[a]Department of Chemistry and Center for Diagnostics and Therapeutics, Georgia State University, Atlanta, GA 30303-3083, USA. [b] Department of Pharmaceutical Organic Chemistry, Faculty of Pharmacy, Mansoura University, Mansoura 35516, Egypt. [c] Master of Pharmaceutical Sciences Program, California North State University, 9700 W Taron Dr., Elk Grove, CA 95757, U.S.A

†These authors contributed equally to this work

Address correspondence to this author

***W. David Wilson Tel: 404-413-5503; Fax: 404-413-5505; Email: wdw@gsu.edu**

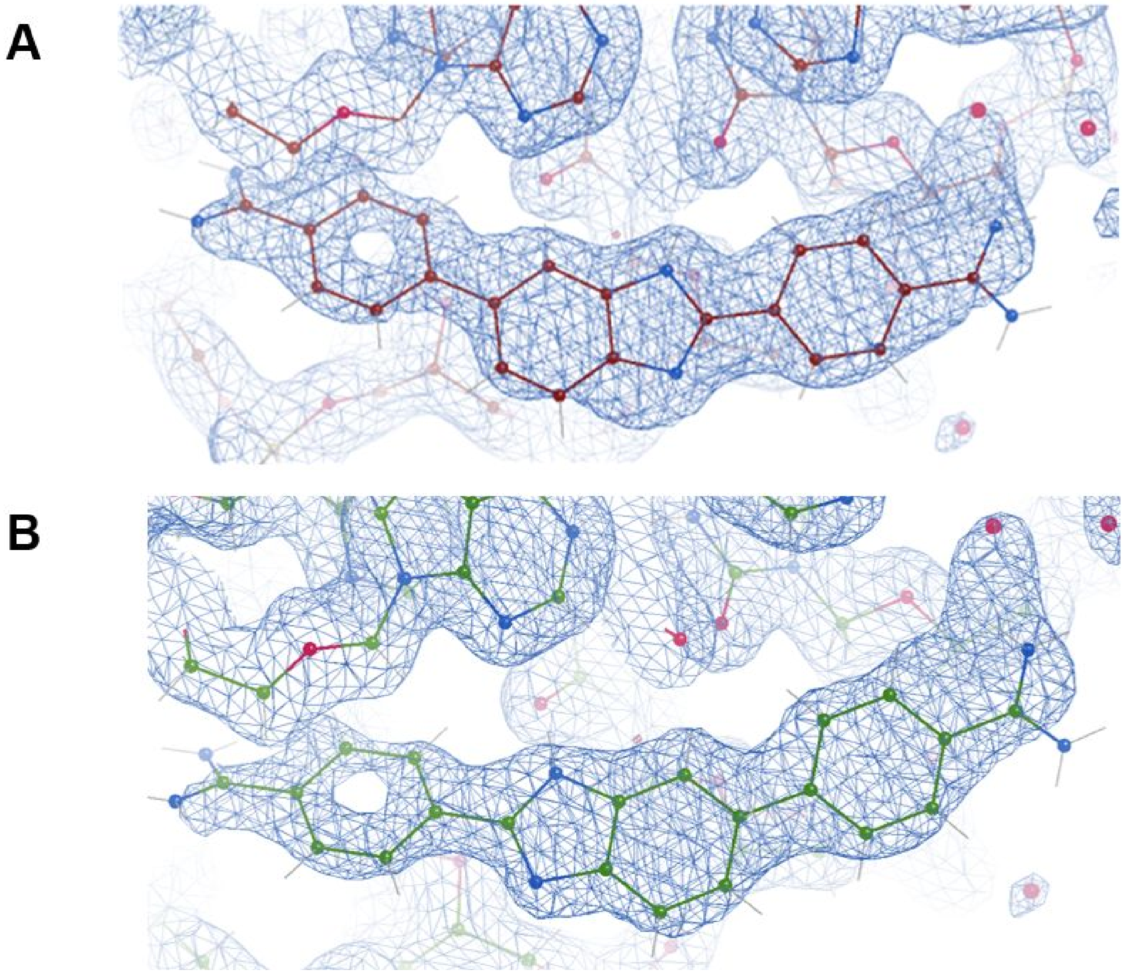

Figure S1: $2F_o$-F_c maps for DB1476 binding in the minor groove of d(5'-CGCGAATTCGCG-3')$_2$.
a) AATT-DB1476-I b) AATT-DB1476-II

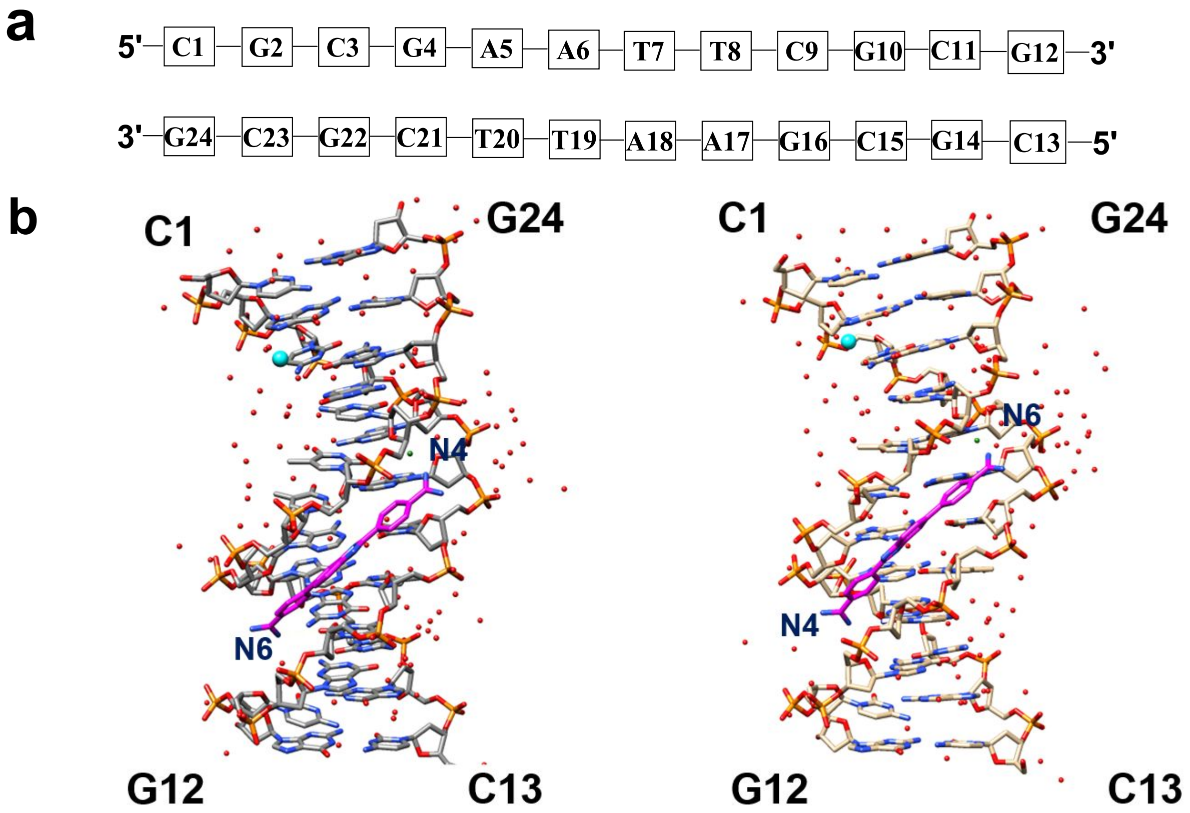

Figure S2: (A) The DNA dodecamer is numbered from 5' C1 to 3' G12 in one strand and from 5' C13 to 3' G24 (B) Structure of **AATT-DB1476-I**. DB1476 is bound to the minor groove in an orientation where the N6 forms H-bond with T20 via an interfacial water-mediated contact. (C) Structure of **AATT-DB1476-II** DB1476 is bound to the minor groove in an orientation where the phenyl-amidine N4 forms an H-bond with T20 via an interfacial water-mediated contact.

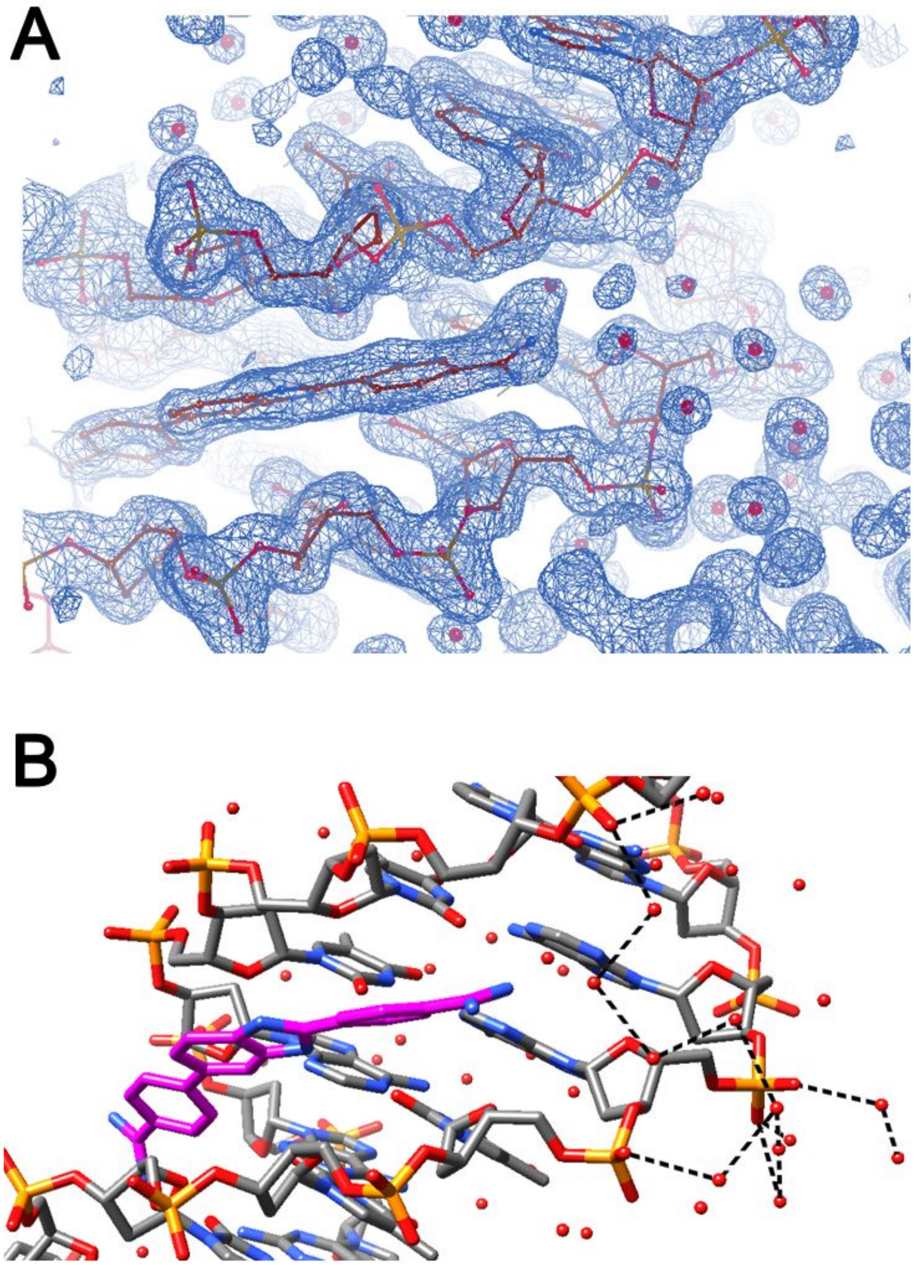

Figure S3: A) 2F$_o$-DF$_c$ map showing the water molecules across the minor groove. B) A model of AATT-DB1476-I showing the water molecule network from the DNA backbone and across the minor groove.

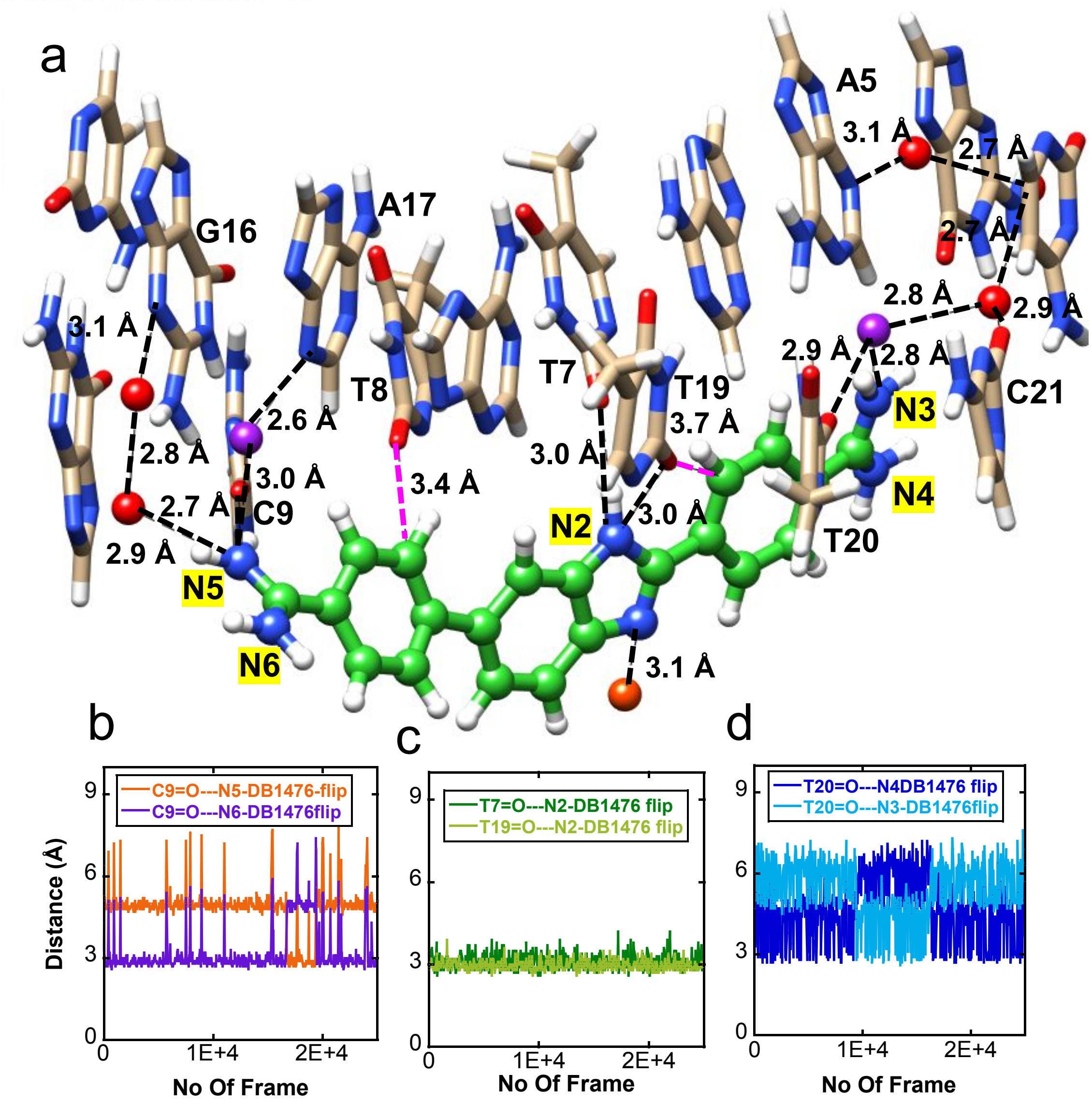

Figure S4: (a) Snapshot of MD simulations of DB1476-flip-d(CGCGAATTCGCG)$_2$ complex; DB1476 bound to the -AATT- site with interfacial water(s)-mediated interaction between the amidines of DB1476 and DNA. The interfacial water (purple, ball, stick, and black dashed lines) completes the bound compound's curvature. DB1476 forms bifurcated direct Bezimidazole-N-H hydrogen bonds with O2s of T7 and T19 (black dashed lines), and two C-H interactions (magenta dashed lines) with DNA bases. Both terminal amidine groups also form extended terminal water (red, ball and stick) networks to stabilize the compound in the minor groove; (b-d) distance plots between the DNA bases and the amidines and benzimidazole-N-H-amidine nitrogen of DB1476. The time interval for each frame in the plot is 20 ps of a 500 ns trajectory (total 25000 frames).

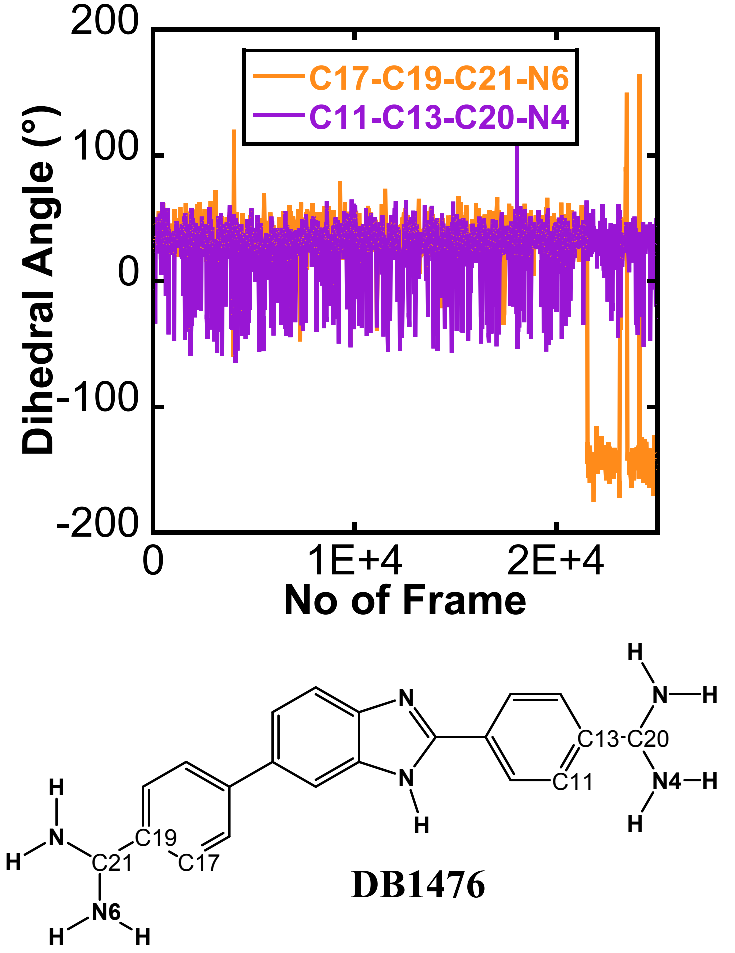

Figure S5: Dihedral Angle plot of DB1476 at -AATT- minor groove During 500 ns MD simulation.

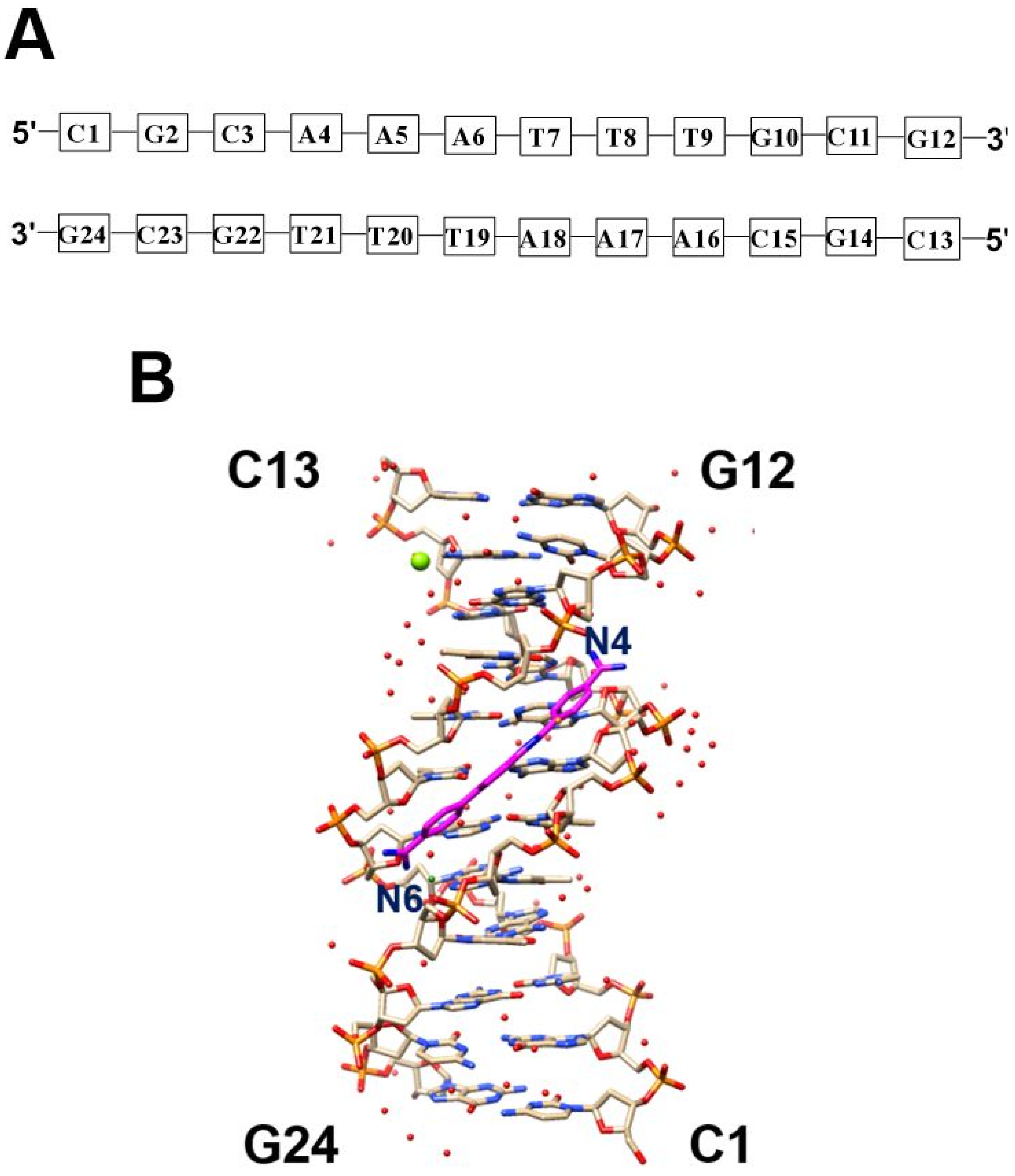

Figure S6: A) The DNA dodecamer is numbered from 5' C1 to 3' G12 in one strand and from 5' C13 to 3' G24 (B) Structure of **AAATTT-DB1476** with surrounding water network. DB1476 is bound to the minor groove of *d*(5'-CGCAAATTTGCG-3')$_2$ in an orientation where A-N6 of the ligand makes an interfacial water-mediated contact with the DNA.

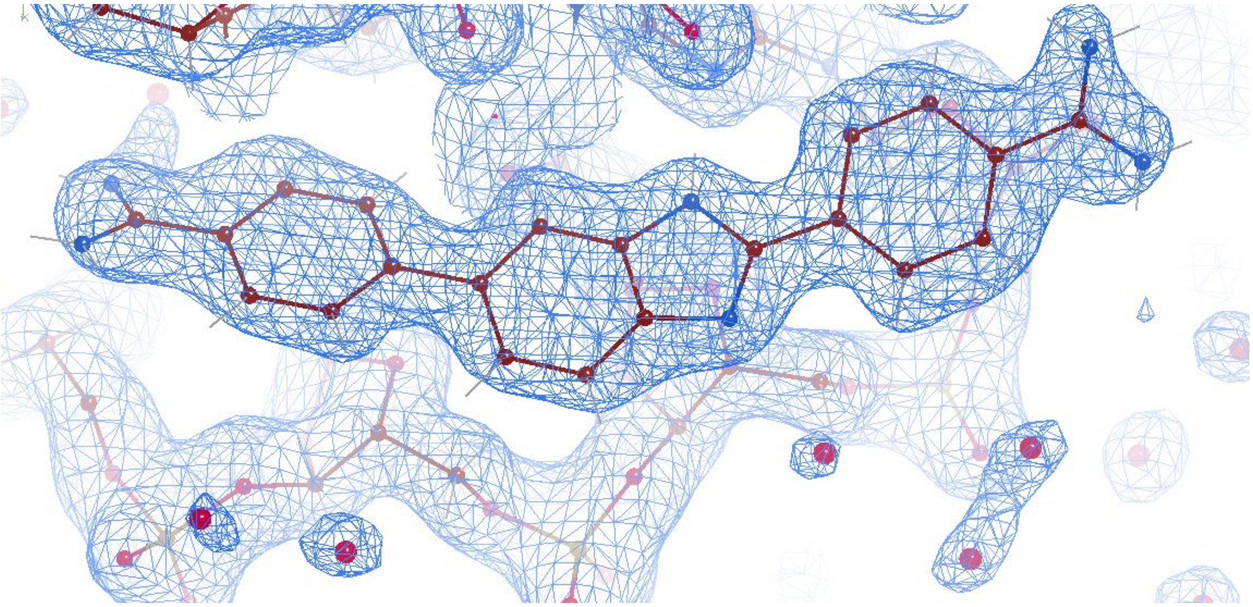

Figure S7: 2F_o-F_c map for DB1476 binding in the minor groove of d(5'-CGCGAATTCGCG-3')$_2$. This is the singular orientation for DB1476 binding in the minor groove.

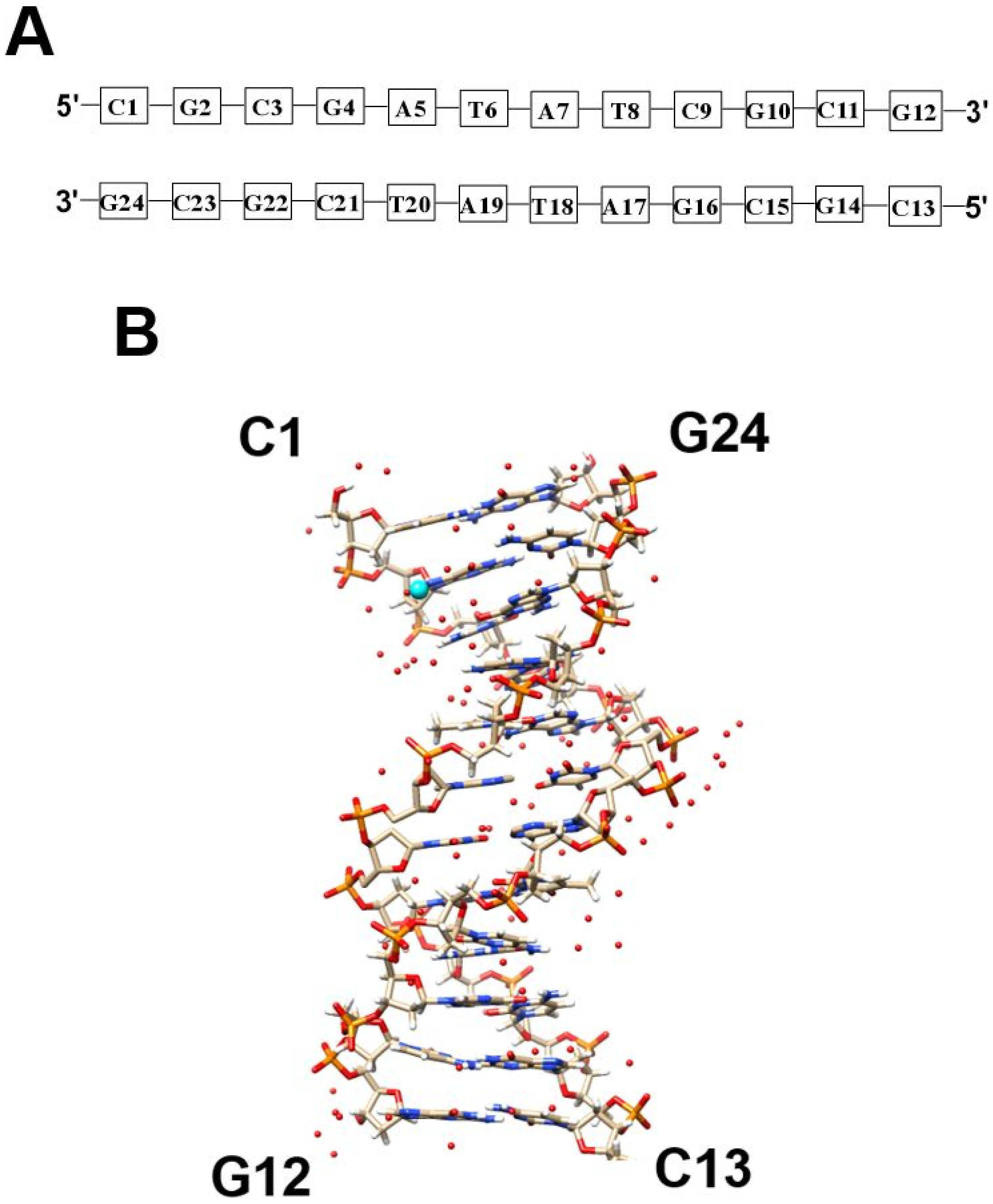

Figure S8: A) The DNA dodecamer is numbered from 5' C1 to 3' G12 in one strand and from 5' C13 to 3' G24 (B) Structure of **ATAT DNA** with surrounding water network.

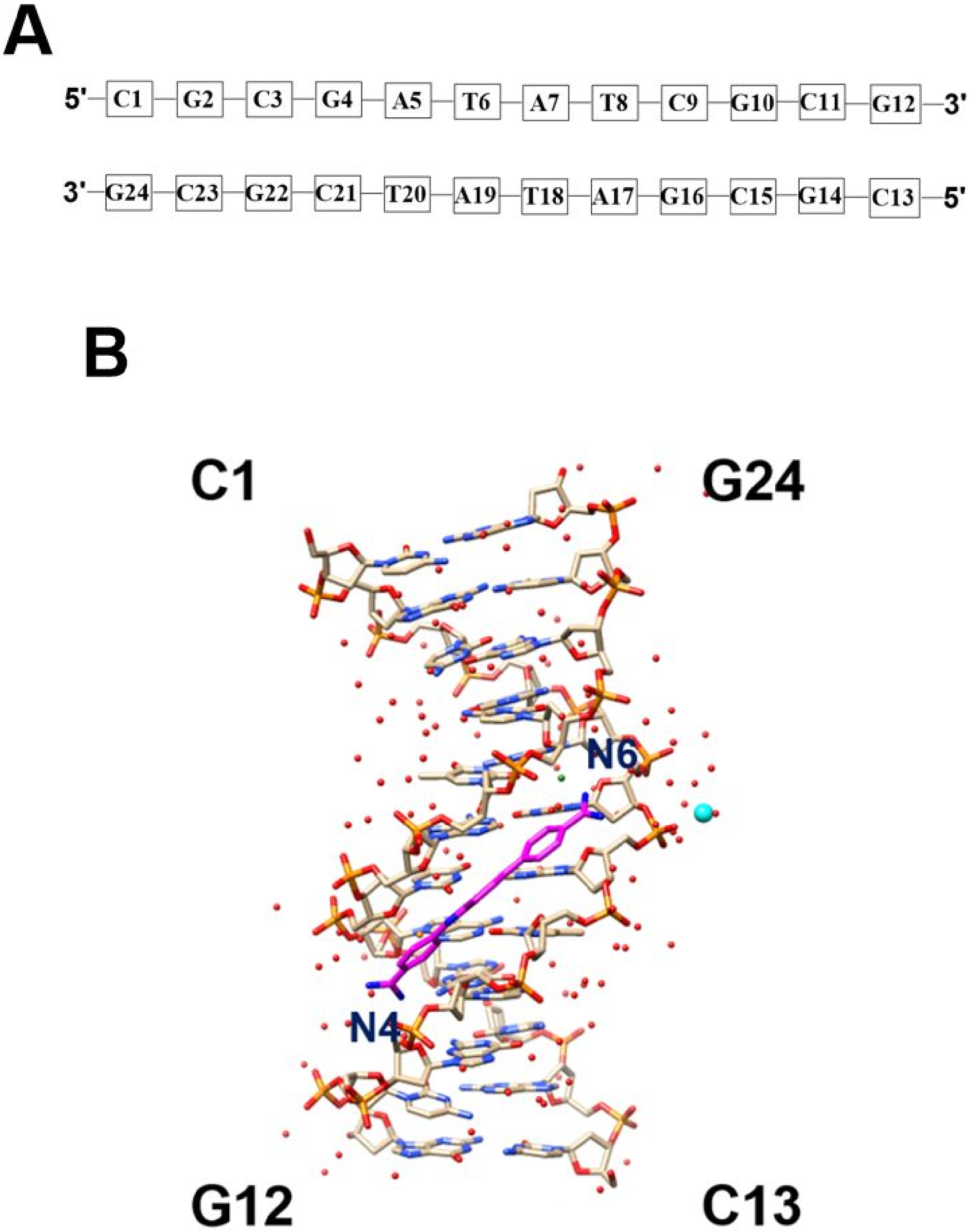
A
5'
C1
G2
C3
G4
A5
T6
A7
T8
C9
G10
C11
G12
3'
3'
G24
C23
G22
C21
T20
A19
T18
A17
G16
C15
G14
C13
5'
B
C1
G24
N6
N4
G12
C13

Figure S9: (A) The DNA dodecamer is numbered from 5' C1 to 3' G12 in one strand and from 5' C13 to 3' G24 (B) Structure of **ATAT-DB1476** with surrounding water network. DB1476 is bound to the minor groove of *d*(5'-CGCGATATCGCG-3')$_2$ in an orientation where A-N6 forms an H-bond via an interfacial water molecule.

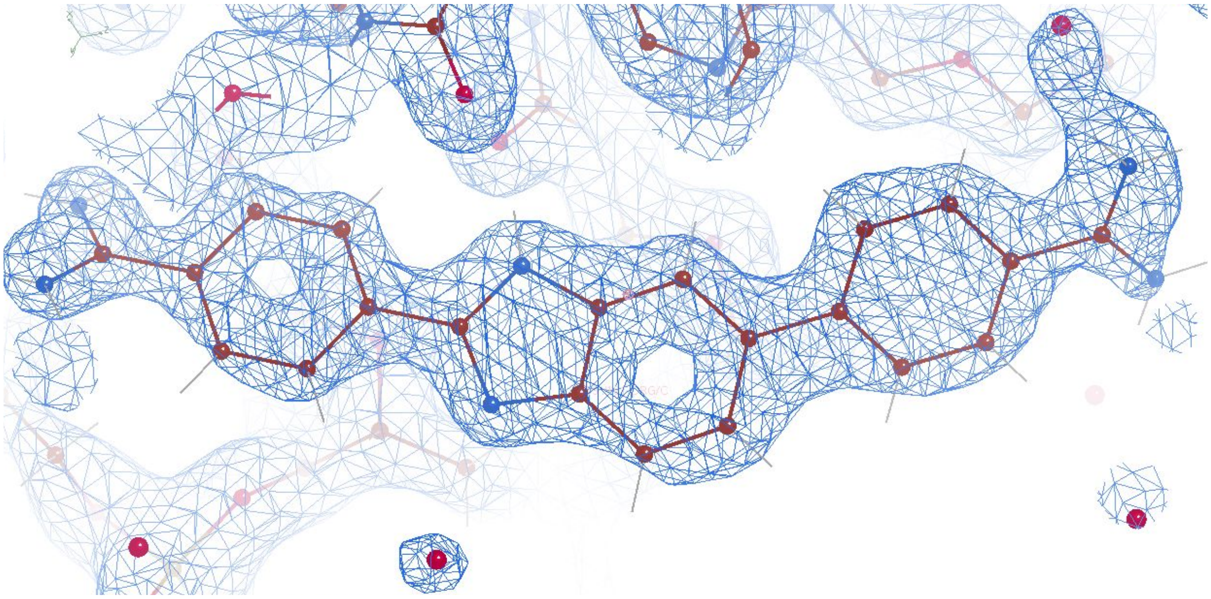

Figure S10: 2F_o-F_c map for DB1476 binding in the minor groove of d(5'-CGCGATATCGCG-3')$_2$. This is the singular orientation for DB1476 binding in the minor groove.

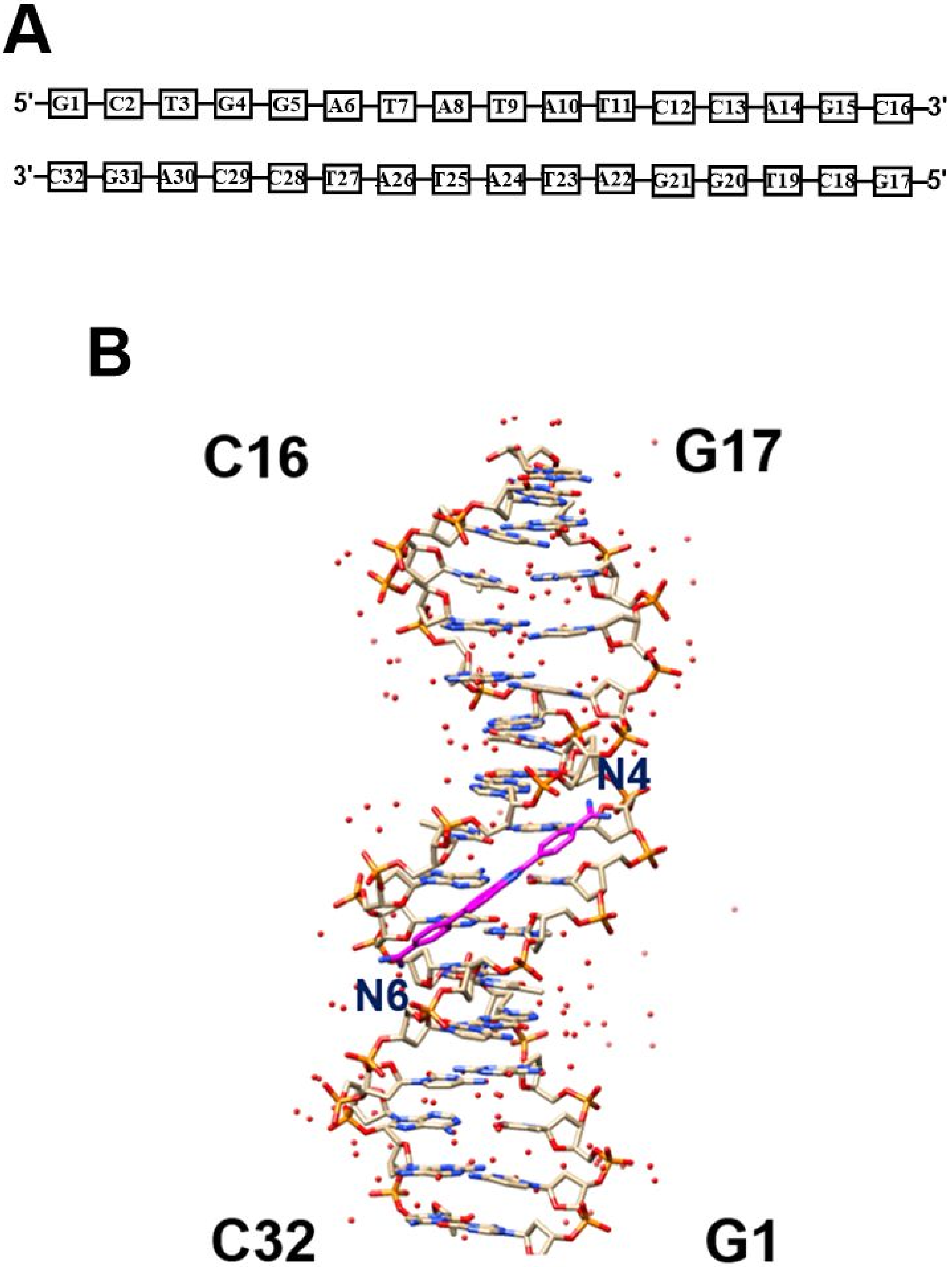

Figure S11: A) The DNA dodecamer is numbered from 5' G1 to 3' C16 in one strand and from 5' G17 to 3' C32 (B) Structure of **ATATAT-DB1476**. DB1476 is bound to the minor groove of *d*(5'-GCTGGATATATCCAGC-3')$_2$ in an orientation where C-N4 of the ligand is positioned to make contact with guanine 5 and adenine 6.

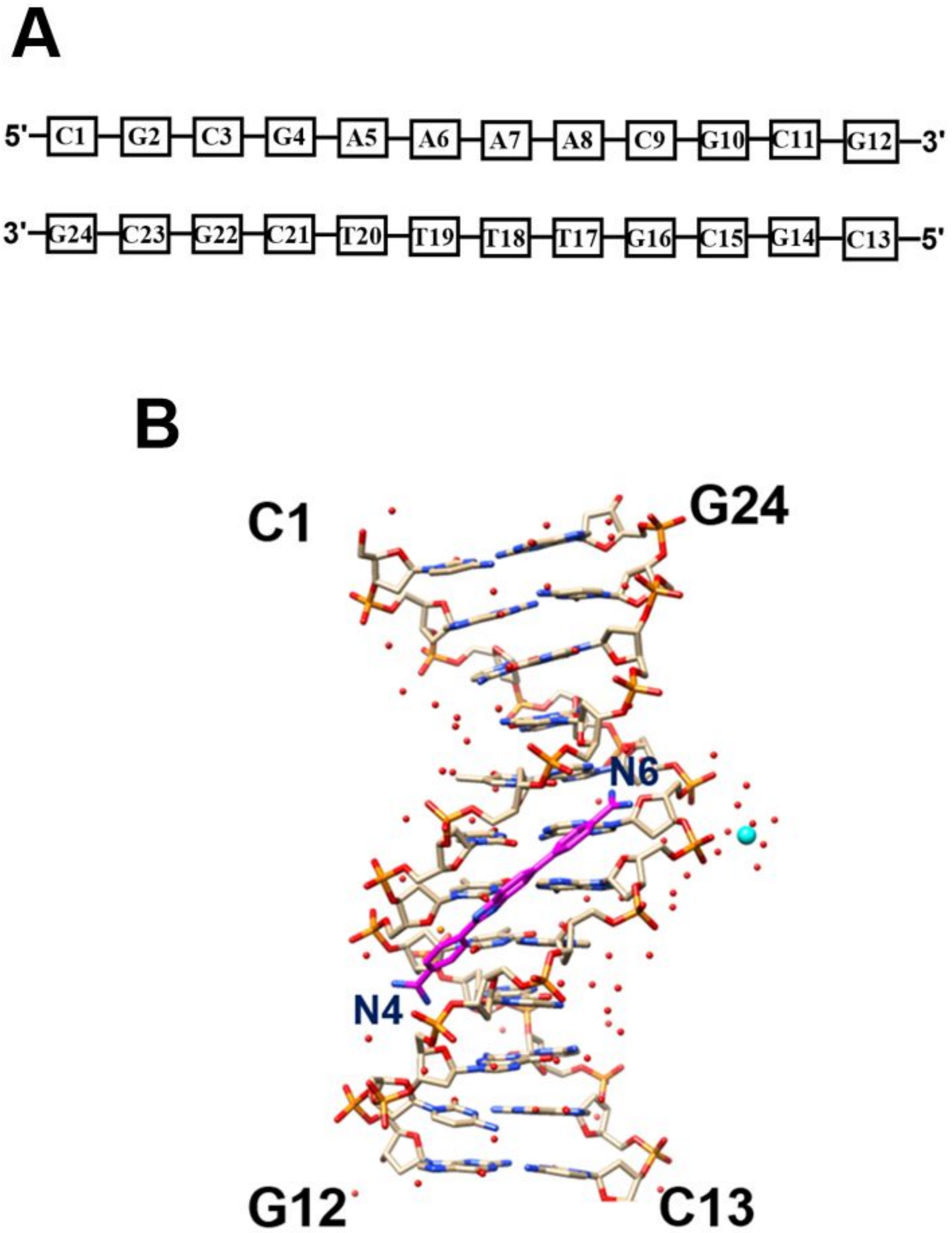

Figure S12: (A) The DNA dodecamer is numbered from 5' C1 to 3' G12 in one strand and from 5' C13 to 3' G24 (B) Structure of **A4-DB1476** with surrounding water network. DB1476 is bound to the minor groove of *d*(5’-CGCGAAAACGCG-3’)$_2$ in an orientation where C-N4 forms a direct hydrogen bond to cytosine 9.

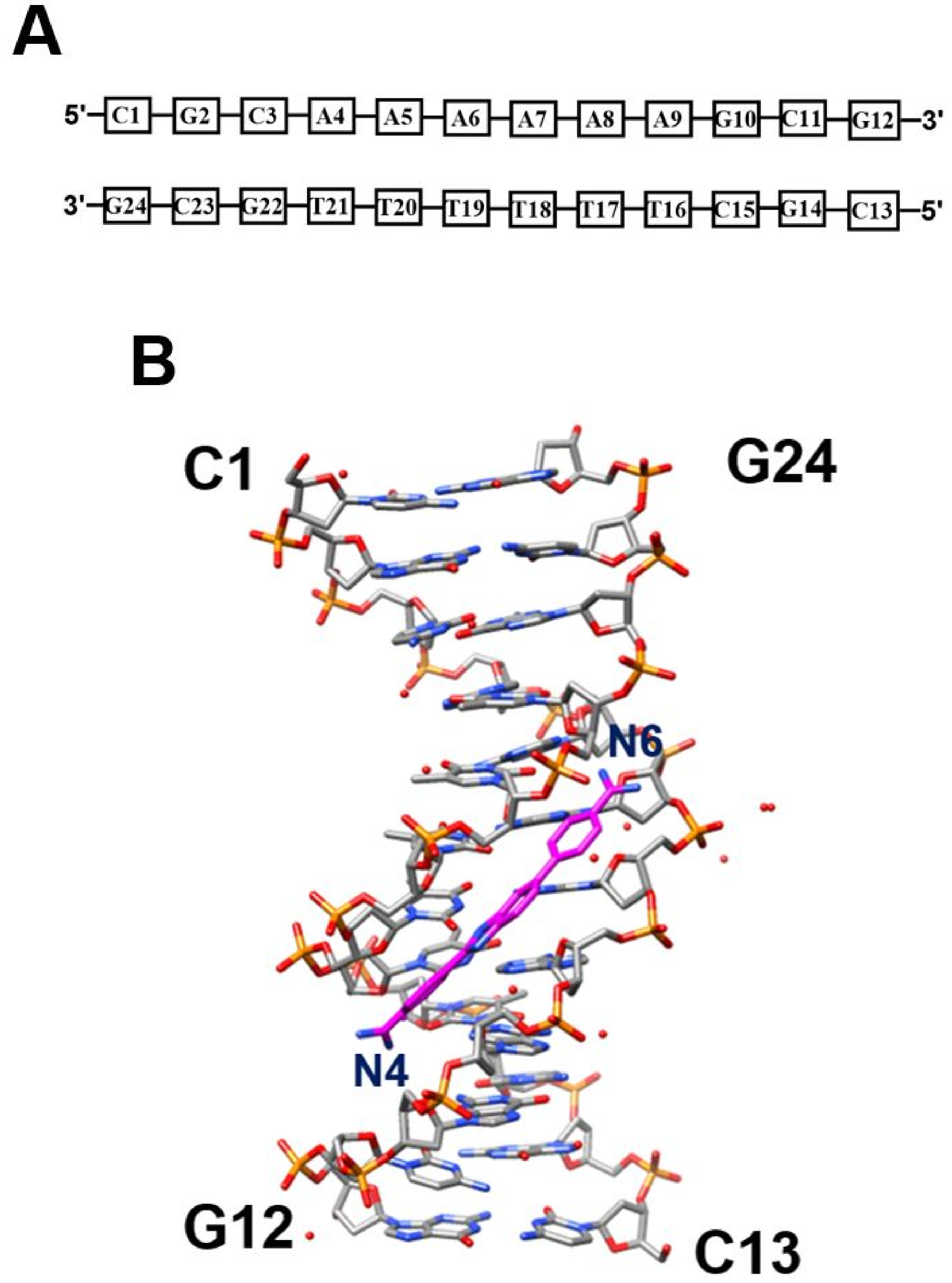

Figure S13: A) The DNA dodecamer is numbered from 5' C1 to 3' G12 in one strand and from 5' C13 to 3' G24 (B) Structure of **A6-DB1476**. DB1476 is bound to the minor groove of *d*(5'-

CGCAAAAAAGCG-3')$_2$ in an orientation where C-N4 of the ligand makes an interfacial water-mediated contact with the DNA.

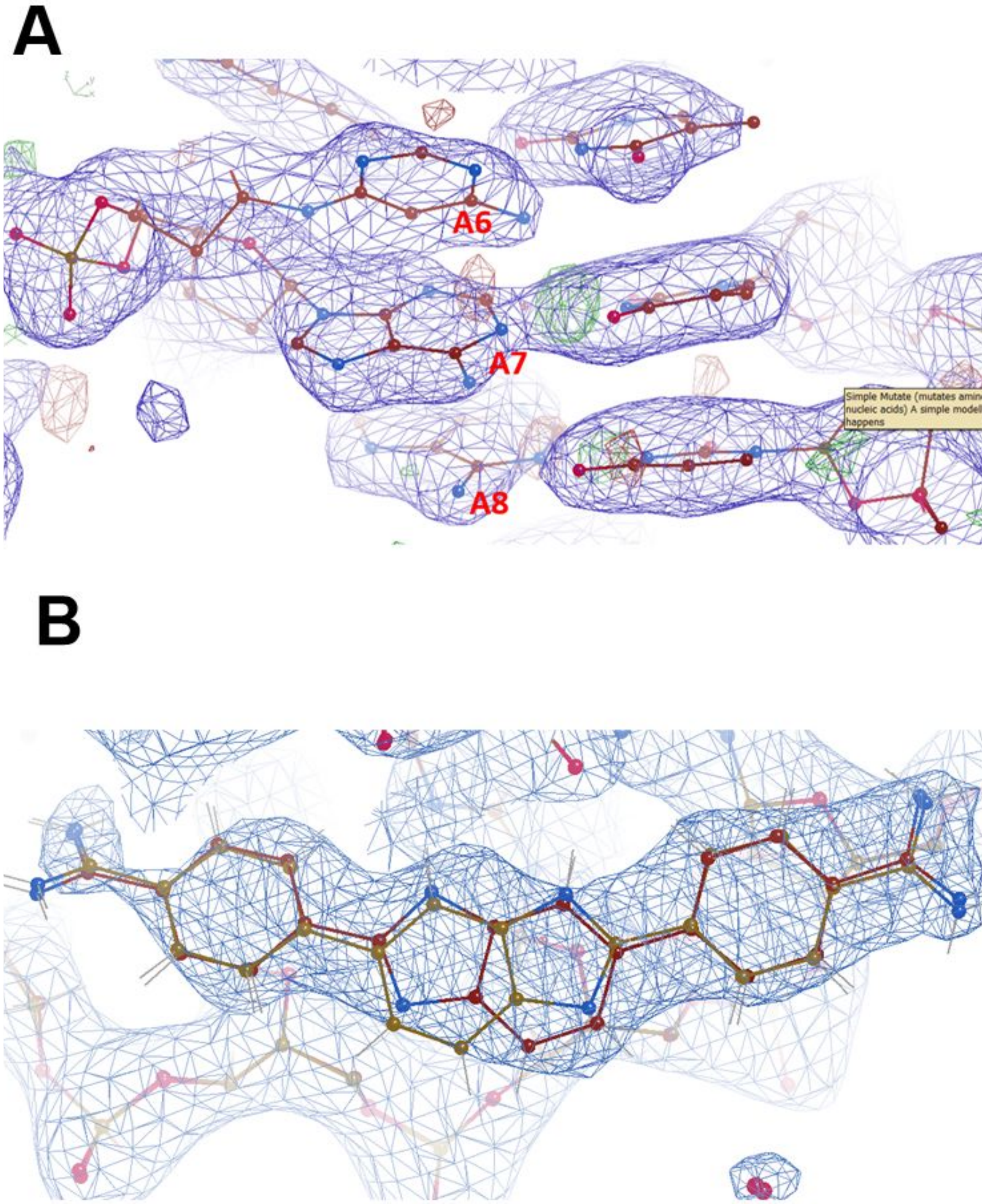

Figure S14: A) Electron density map distortions can clearly be seen between A7 and A8 (B) 2FoFc MAP of DB1476 orientations in the minor groove of -A6- structure. A6-DB1476-I is in yellow. A6-DB1476-II is in red.

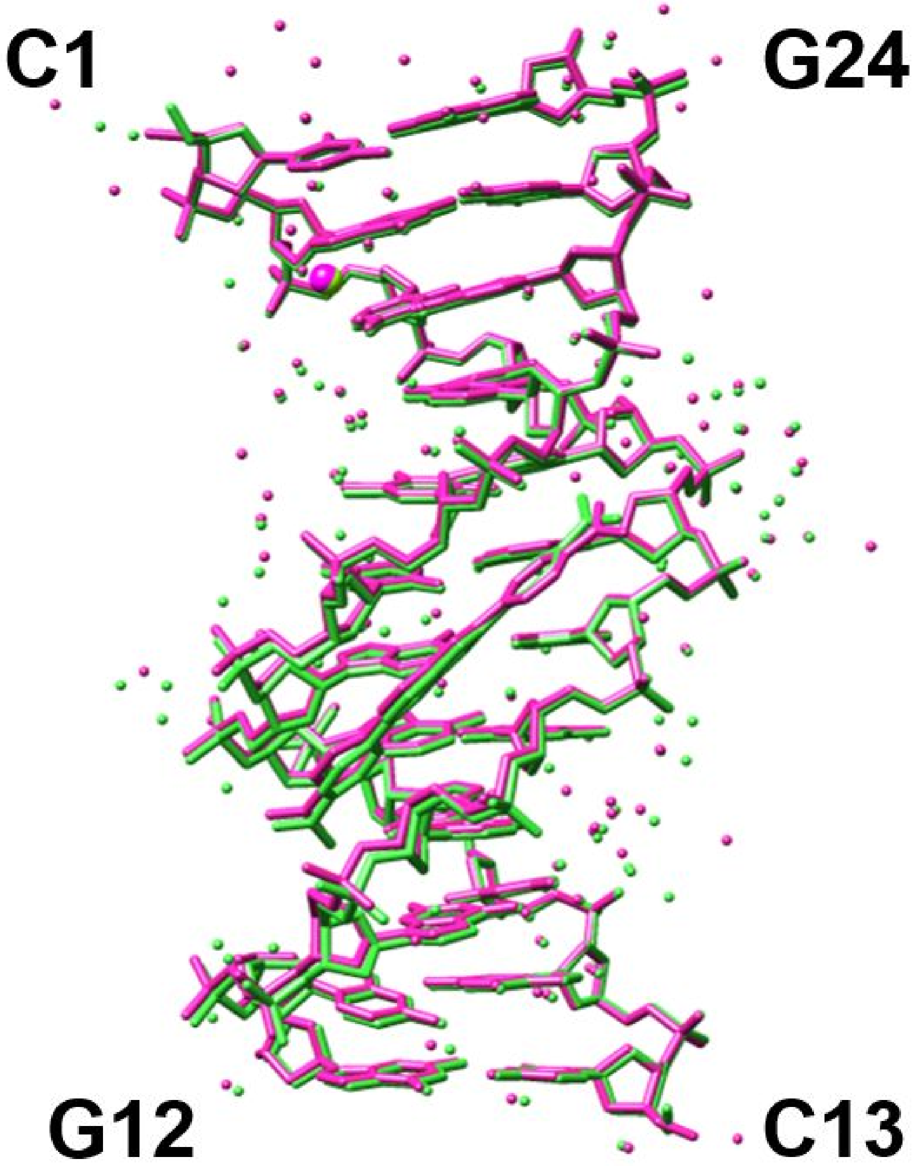

Figure S15: Overlay of AATT-DB1476-I and AATT-DB1884 complexes. Both complexes follow the same dodecamer numbering as Figure S2.

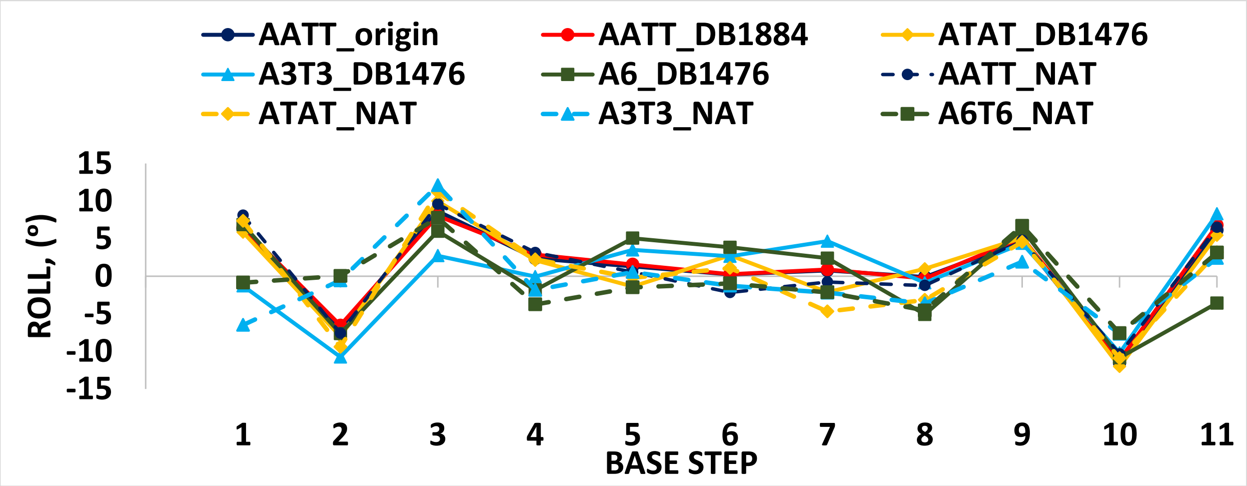

Figure S16: A) Plot of base step roll for all the studied DNA-DB1476 complexes. AT DNA-DB1476 complex in solid line, native in dashed line. No significant difference in between DNA-DB176 complexes and their native structures.

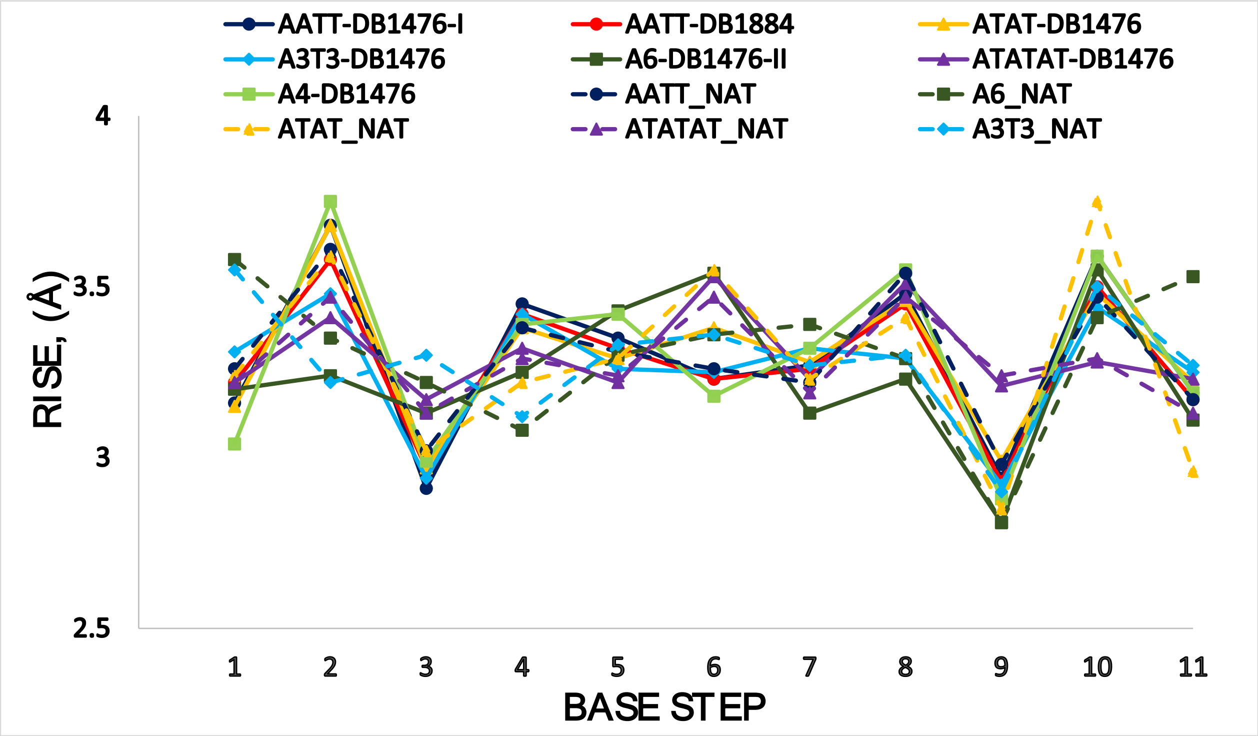

Figure S17: A) Plot of base step rise for all the studied DNA-DB1476 complexes. AT DNA-DB1476 complex in solid line, native in dashed line. No significant difference in between DNA-DB176 complexes and their native structures.

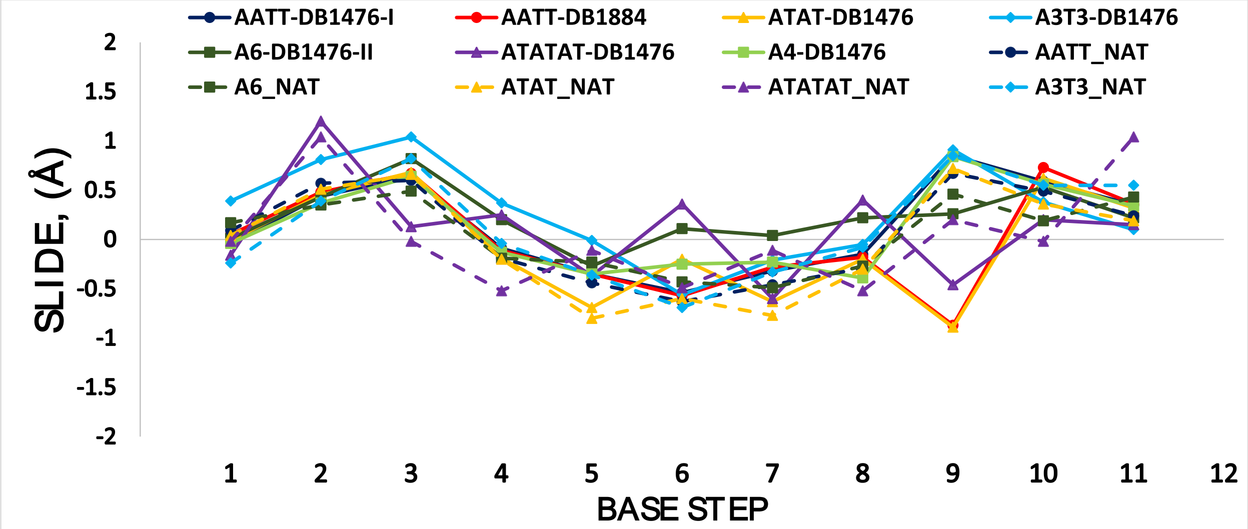

Figure S18: A) Plot of base step slide for all the studied DNA-DB1476 complexes. AT DNA-DB1476 complex in solid line, native in dashed line. No significant difference in between DNA-DB176 complexes and their native structures.

Table S1: Crystallization Setup. I) Experiment set-up is described II) Crystallization conditions used are stated.

I.

Crystallization DNA-Ligand	Annealing	DNA (mM)	Compund (mM)	Condition
5'-GAATTC-3'-DB1476/I	20mM Tris HCL PH 7.4	1.00	1.60	9
5'-GAATTC-3'-DB1476/II	20mM Tris HCL PH 7.4	1.00	1.60	9
5'-GAATTC-3'-DB1884	20mM Tris HCL PH 7.4	1.00	1.60	14
5'-GATATC-3'-DB1476	20mM Tris HCL PH 7.4	0.80	1.76	16
5'-AAATTT-3'-DB1476	20mM Tris HCL PH 7.4	0.80	1.92	16
5'-AAAAAA-3'- DB1476	20mM Tris HCL PH 7.4	0.80	2.00	8
5'-GAAAAC-3'-DB1476	20Mm Tris HCL PH 7.4	1.00	2.00	9
5'-GAATTC-3'	20mM Tris HCL PH 7.4	1.00	0.00	10
5'-GATATC-3'	20mM Tris HCL PH 7.4	1.00	0.00	9
5'-AAATTT-3'	20mM Tris HCL PH 7.4	1.00	0.00	9
5'-AAAAAA-3'	20mM Tris HCL PH 7.4	0.80	0.00	10
5'-ATATAT-3'-DB1476	7.5mm HEPES PH 6.6	1.00	3.00	X

II.

Conditions

	Precipitant	Buffer	Polyamine	Monovalent Ion	Divalent Ion
8	10% v/v (±)-2-methyl-2,4-pentanediol (MPD)	0.04 M Sodium cacodylate trihydrate PH 6.0	0.012 M Spermine tetrachloride	0.080 M NaCl	None
9	10% v/v (±)-2-methyl-2,4-pentanediol (MPD)	0.04 M Sodium cacodylate trihydrate PH 6.0	0.012 M Spermine tetrachloride	0.080 M NaCl/0.012M KCl	0.020 M $MgCl.6H_20$
10	10% v/v (±)-2-methyl-2,4-pentanediol (MPD)	0.04 M Sodium cacodylate trihydrate PH 6.0	0.012 M Spermine tetrachloride	0.012 M NaCl/0.080M KCl	None
11	10% v/v (±)-2-methyl-2,4-pentanediol (MPD)	0.04 M Sodium cacodylate trihydrate PH 6.0	0.012 M Spermine tetrachloride	0.080 M KCl	0.020 M $BaCl_2$
14	10% v/v (±)-2-methyl-2,4-pentanediol (MPD)	0.04 M Sodium cacodylate trihydrate PH 7.0	0.012 M Spermine tetrachloride	0.080 M KCl	0.020 M $MgCl.6H_20$
15	10% v/v (±)-2-methyl-2,4-pentanediol (MPD)	0.04 M Sodium cacodylate trihydrate PH 7.1	0.012 M Spermine tetrachloride	0.080 M KCl	None
16	10% v/v (±)-2-methyl-2,4-pentanediol (MPD)	0.04 M Sodium cacodylate trihydrate PH 7.0	0.012 M Spermine tetrachloride	0.080 M NaCl	0.020 M $MgCl.6H_20$
17	10% v/v (±)-2-methyl-2,4-pentanediol (MPD)	0.04 M Sodium cacodylate trihydrate PH 7.0	0.012 M Spermine tetrachloride	0.080 M NaCl	None
X	40% PEG 200	10mM HEPES	---	---	600Mm CaCl2

Table S2: Collection Source and Details.

CRYSTAL	FACILITY	BEAMLINE	DETECTOR	WAVELENGTH(Å)
**AATT-DB1476, AATT-1884, AATT_N**	APS Argonne, IL	22-BM	Rayonix MX300-HS CCD	1
**ATAT-DB1476**	ALS Berkeley, CA	BL-8.2.2	Rayonix MX300-HS CCD	0.97
**A3T3-DB1476, A6-DB1476, ATAT_N, A3T3_N, A6_N, AATT_N, A4-DB1476, ATATAT-DB1476, ATATAT_N, ATATAT_DB1476**	NSLS-II Upton, NY	AMX 17-ID-1	Eiger9M	1

***N : Native**

Table S3: X-ray crystallographic information for DNA-ligand complexes

	AATT-DB1476-I	AATT-DB1476-II	AATT-DB1884	ATAT-DB1476
**PDB Accession ID**	8EC1	8ED6	8EDB	8EDA
**Data Collection**				
Space group	P 2_1 2_1 2_1	P 2_1 2_1 2_1	P 2_1 2_1 2_1	P 2_1 2_1 2_1
Cell dimensions				
a, *b*, *c* (Å)	25.8, 40.1, 65.5	25.8, 40.1, 65.5	25.8, 39.8, 65.4	25.7, 39.9, 66.4
a, b, g (°)	90, 90, 90	90, 90, 90	90, 90, 90	90, 90, 90
Resolution (Å)	34.2 - 1.63	34.2 - 1.63	21.6 - 1.55	34.2 - 1.63
R_{merge}	8.541e-18 (8.926e-18)	8.541e-18 (8.926e-18)	0.0148 (0.0894)	1.296e-17 (2.122e-17)
I / s*I*	27.7 (4.42)	27.7 (4.42)	17.92 (5.30)	21.73 (4.21)
Completeness (%)	99.0 (92.7)	99.0 (92.7)	99.5 (99.2)	99.2(95.1)
Unique Reflections	8886 (807)	8886 (807)	10249 (1004)	8949 (840)
Multiplicity	1.20 (1.10)	1.20 (1.10)	2.0 (2.0)	1.2 (1.1)
**Refinement**				
Resolution (Å)	1.63	1.63	1.55	1.63
No. reflections	8886 (796)	8856 (798)	10204 (997)	8949 (840)
R_{work} / R_{free}	0.184/0.227	0.183/0.222	0.22/0.26	0.183/0.201
**No. atoms**				
DNA	486	486	486	486
Ligand/ion	48	48	49	48
Water	105	109	105	118
**Average B-factor**	27.3	27.2	23.2	26.0
**R.m.s. deviations**				
Bond angles (°)	1.49	1.48	1.58	1.44

	AAATTT-DB1476	A6-DB1476-I	A6-DB1476-II	A4-DB1476	ATATAT-DB1476
**PDB Accession ID**	8FDQ	8F1S	8F1V	8FDR	8FB4
**Data Collection**					
Space group	P 2_1 2_1 2_1	P 2_1 2_1 2_1	P 2_1 2_1 2_1	P 2_1 2_1 2_1	P 3_1 2 1
Cell dimensions					
a, *b*, *c* (Å)	24.4, 38.4, 60.8	23.1, 41.5, 64.0	23.1, 41.5, 64.0	25.8, 39.8, 66.5	38.7, 38.7, 160.4
a, b, g (°)	90, 90, 90	90, 90, 90	90, 90, 90	90, 90, 90	90, 90, 120
Resolution (Å)	22.6 - 1.54	23.3 - 2.1	23.3 - 2.1	24.02 - 1.55	25.7 - 1.49
R_{merge}	0.01743 (0.1699)	0.02185 (0.1372)	0.02185 (0.1372)	0.009282 (0.1607)	0.01058 (0.1428)
I / s*I*	14.06 (3.38)	9.71 (2.69)	9.71 (2.69)	22.3 (3.73)	31.2 (5.18)
Completeness (%)	99.3 (98.7)	99.6 (99.5)	99.6 (99.5)	99.5 (99.5)	99.2 (99.0)
Unique Reflections	9011 (881)	4201 (405)	4201 (405)	10434 (1011)	23742 (2299)
Multiplicity	2.0 (2.0)	2.0 (2.0)	2.0 (2.0)	2.0 (2.0)	2.0 (2.0)
**Refinement**					
Resolution (Å)	1.54	2.10	2.10	1.55	1.49
No. reflections	8994 (879)	4185 (403)	4186 (403)	10434 (1007)	23742 (2294)
R_{work} / R_{free}	0.204/0.242	0.260/0.311	0.277/0.327	0.220/0.247	0.267/0.300
**No. atoms**					
DNA	486	486	486	486	975
Ligand/ion	48	48	48	48	107
Water	72	19	14	75	160
**Average B-factor**	25.7	48.8	45.1	29.2	30.8
**R.m.s. deviations**					
Bond angles (°)	1.45	1.31	1.32	1.31	1.49

	AATT_NAT	A6_NAT	AAATTT_NAT	ATAT_NAT	ATATAT_NAT
**PDB Accession ID**	8F2W	8F2O	8FDP	8F2Y	8F94
**Data Collection**					
Space group	P 2_1 2_1 2_1	P 2_1 2_1 2_1	P 2_1 2_1 2_1	P 2_1 2_1 2_1	R 3 2 H
Cell dimensions					
a, *b*, *c* (Å)	25.6, 40.1, 66.1	25.7, 41.6, 65.2	25.0, 41.5, 65.4	25.5, 40.3, 66.7	38.8, 38.8, 159.8
a, b, g (°)	90, 90, 90	90, 90, 90	90, 90, 90	90, 90, 90	90, 90, 120
Resolution (Å)	34.29 - 1.3	23.88.1 - 2.23	23.3 - 1.88	25.7 - 1.78	26.6 - 1.08
R_{merge}	0.06401 (0.1921)	0.008562 (0.1661)	0.01009 (0.1474)	0.0510 (0.5000)	0.01722 (0.148)
I / s*I*	16.74(5.9)	24.0 (3.2)	25.0 (3.8)	11.2 (3.1)	25.1 (6.61)
Completeness (%)	95.2 (96.5)	99.3 (100.0)	99.5 (99.7)	99.6 (99.7)	97.9 (92.9)
Unique Reflections	16736 (1649)	3702 (374)	5921 (577)	7017 (692)	20060 (1882)
Multiplicity	1.9 (2.0)	2.0 (2.0)	2.0 (2.0)	2.0 (2.0)	2.0 (2.0)
**Refinement**					
Resolution (Å)	1.3	2.23	1.88	1.78	1.08
No. reflections	16736 (1649)	3703 (375)	5921 (576)	7017 (689)	20059 (1865)
R_{work} / R_{free}	0.187/0.215	0.252/0.285	0.246/0.276	0.209/0.225	0.200/0.222
**No. atoms**					
DNA	486	486	486	486	325
Ligand/ion	1	0	0	1	9
Water	135	5	28	95	97
**Average B-factor**	20.2	62.1	39.4	23.5	30.8
**R.m.s. deviations**					
Bond angles (°)	1.44	1.32	1.25	1.4	2.11

Table S4: Biosensor-SPR Equilibrium Dissociation Constants (K_D, nM) of DB1476 with Pure A·T sequences

DNA	DB1476 (K_D nM)
AATT	1
AAATTT	0.5
ATAT	12
A6	2
A4	15
ATATAT	16

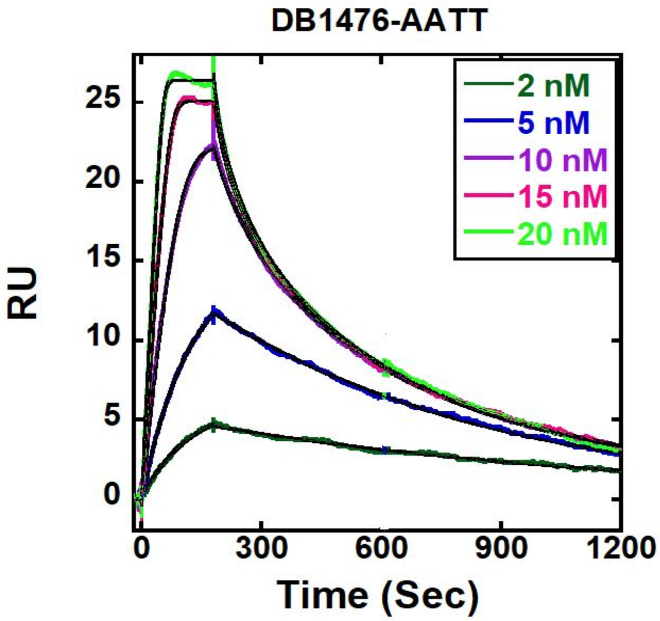

[a] The listed binding affinities are an average of two independent experiments carried out with two different sensor chips, and the values are reproducible within 10% experimental errors. The experiments were conducted in Tris-HCl buffer (50 mM Tris-HCl, 100 mM NaCl, 1 mM EDTA, pH 7.4) at 25 °C.

Table S5: Helical Parameters for AATT-DB1476-I

Local base-pair parameters

bp	Shear	Stretch	Stagger	Buckle	Propeller	Opening
1 C-G	0.36	-0.14	0.22	5.15	-16.48	0.39
2 G-C	-0.22	-0.22	0.51	7.74	-12.53	-2.38
3 C-G	0.18	-0.18	0.32	-6.07	-5.80	-0.21
4 G-C	-0.15	-0.05	0.08	12.95	-8.38	0.50
5 A-T	0.09	-0.07	-0.04	6.45	-18.08	1.28
6 A-T	-0.01	-0.14	0.02	0.99	-18.64	4.94
7 T-A	-0.02	-0.16	0.08	-0.16	-18.34	5.85
8 T-A	-0.11	-0.21	-0.13	-3.56	-18.13	5.43
9 C-G	0.13	-0.14	0.02	-11.66	-6.31	-1.94
10 G-C	-0.14	-0.13	0.32	8.89	-5.66	3.64
11 C-G	0.14	-0.18	0.45	0.11	-19.79	-2.72
12 G-C	-0.25	-0.12	0.33	1.50	-8.22	-0.84
ave.	-0.00	-0.15	0.18	1.86	-13.03	1.16

Local base-pair step parameters

step	Shift	Slide	Rise	Tilt	Roll	Twist
1 CG/CG	-0.06	-0.04	3.16	-2.89	6.34	33.42
2 GC/GC	0.59	0.44	3.68	1.95	-6.92	42.75
3 CG/CG	-0.28	0.64	2.91	2.98	8.69	26.53
4 GA/TC	-0.18	-0.09	3.45	-0.40	2.38	38.02
5 AA/TT	0.19	-0.35	3.35	-0.51	1.33	35.07
6 AT/AT	0.13	-0.54	3.23	-0.23	0.25	31.51
7 TT/AA	-0.06	-0.32	3.27	2.60	0.77	34.13
8 TC/GA	-0.25	-0.15	3.48	-0.76	-0.09	41.19
9 CG/CG	0.51	0.85	2.94	-3.94	4.78	26.87
10 GC/GC	-1.16	0.59	3.59	-3.86	-11.54	42.74
11 CG/CG	-0.07	0.35	3.19	0.78	6.13	32.90
ave.	-0.06	0.12	3.30	-0.39	1.10	35.01

Table S6: Helical Parameters for AATT-DB1476-II

Local base-pair parameters

bp	Shear	Stretch	Stagger	Buckle	Propeller	Opening
1 C-G	0.34	-0.14	0.20	4.87	-15.93	0.45
2 G-C	-0.21	-0.23	0.47	6.99	-11.83	-2.65
3 C-G	0.17	-0.21	0.32	-5.81	-5.42	-0.61
4 G-C	-0.16	-0.03	0.08	12.68	-7.99	0.40
5 A-T	0.10	-0.08	-0.04	6.00	-17.68	1.96
6 A-T	-0.01	-0.10	0.02	0.98	-17.92	4.84
7 T-A	-0.04	-0.12	0.02	-0.24	-17.88	6.46
8 T-A	-0.06	-0.22	-0.12	-3.90	-17.29	5.51
9 C-G	0.10	-0.16	0.02	-12.14	-6.80	-1.96
10 G-C	-0.17	-0.13	0.30	8.19	-5.53	3.52
11 C-G	0.16	-0.18	0.47	0.35	-21.21	-2.43
12 G-C	-0.22	-0.12	0.43	2.41	-7.72	-1.30
ave.	0.00	-0.14	0.18	1.70	-12.77	1.18

Local base-pair step parameters

step	Shift	Slide	Rise	Tilt	Roll	Twist
1 CG/CG	-0.06	-0.01	3.17	-2.57	6.65	33.59
2 GC/GC	0.58	0.42	3.66	1.52	-6.91	42.36
3 CG/CG	-0.25	0.64	2.93	3.20	8.33	26.71
4 GA/TC	-0.14	-0.09	3.44	-0.34	2.90	38.21
5 AA/TT	0.15	-0.34	3.35	-0.69	1.11	34.86
6 AT/AT	0.16	-0.52	3.24	0.29	0.12	31.54
7 TT/AA	-0.07	-0.29	3.28	2.18	1.13	34.52
8 TC/GA	-0.27	-0.17	3.48	-0.49	-0.30	40.62
9 CG/CG	0.53	0.86	2.94	-3.95	4.90	26.85
10 GC/GC	-1.16	0.62	3.57	-3.73	-11.56	43.17
11 CG/CG	-0.07	0.32	3.18	-0.04	5.92	33.18
ave.	-0.05	0.13	3.29	-0.42	1.12	35.06

Table S7: Helical Parameters for AAATTT-DB1476

Local base-pair parameters

bp	Shear	Stretch	Stagger	Buckle	Propeller	Opening
1 C-G	0.10	-0.16	0.34	-2.16	-4.38	-2.02
2 G-C	-0.05	-0.27	0.37	2.04	-16.97	-3.96
3 C-G	0.01	-0.10	0.05	-2.19	-4.12	1.19
4 A-T	0.07	-0.01	0.11	18.71	-11.34	1.54
5 A-T	0.19	-0.13	0.08	10.14	-19.04	1.27
6 A-T	-0.01	-0.14	0.21	3.15	-21.90	7.38
7 T-A	-0.11	-0.16	0.23	-1.17	-18.54	7.55
8 T-A	-0.08	-0.15	0.07	-10.45	-19.27	3.40
9 T-A	-0.09	-0.00	-0.14	-14.73	-12.13	-3.67
10 G-C	-0.04	-0.10	0.19	5.72	-6.14	0.61
11 C-G	0.15	-0.24	0.28	1.03	-17.98	-3.02
12 G-C	-0.23	-0.18	0.24	-1.58	-11.19	-0.94
ave.	-0.01	-0.14	0.17	0.71	-13.58	0.78

Local base-pair step parameters

step	Shift	Slide	Rise	Tilt	Roll	Twist
1 CG/CG	-0.07	0.39	3.31	0.61	-1.27	36.21
2 GC/GC	0.63	0.81	3.48	3.21	-10.74	42.31
3 CA/TG	-0.24	1.04	2.94	1.23	2.74	28.58
4 AA/TT	-0.35	0.37	3.42	-1.58	-0.05	40.88
5 AA/TT	0.34	-0.01	3.26	-1.78	3.51	37.29
6 AT/AT	0.25	-0.56	3.25	0.18	2.66	30.23
7 TT/AA	-0.14	-0.21	3.32	2.66	4.68	37.61
8 TT/AA	-0.42	-0.05	3.29	2.83	-0.87	39.35
9 TG/CA	0.74	0.91	2.92	-4.71	4.38	28.31
10 GC/GC	-1.08	0.38	3.44	-3.57	-10.20	41.76
11 CG/CG	-0.34	0.10	3.25	-0.93	8.31	33.05
ave.	-0.06	0.29	3.26	-0.17	0.29	35.96

Table S8: Helical Parameters for ATAT-DB1476

Local base-pair parameters

bp	Shear	Stretch	Stagger	Buckle	Propeller	Opening
1 C-G	0.36	-0.19	0.13	4.46	-14.28	-0.68
2 G-C	-0.13	-0.20	0.51	9.28	-10.44	-2.31
3 C-G	0.24	-0.18	0.33	-4.70	-8.69	0.84
4 G-C	-0.25	-0.09	0.12	11.77	-9.24	-0.03
5 A-T	0.17	-0.11	0.08	6.12	-17.79	-0.26
6 T-A	-0.05	-0.10	0.07	4.88	-11.77	3.68
7 A-T	-0.01	-0.16	0.09	-0.56	-13.42	4.60
8 T-A	-0.03	-0.20	-0.14	-0.99	-16.90	5.02
9 C-G	0.16	-0.09	-0.01	-8.94	-8.29	-1.58
10 G-C	-0.13	-0.18	0.34	9.98	-6.22	2.17
11 C-G	0.11	-0.20	0.30	3.35	-21.93	-2.88
12 G-C	-0.21	-0.12	0.54	5.05	-8.08	-1.65
ave.	0.02	-0.15	0.20	3.31	-12.25	0.58

Local base-pair step parameters

step	Shift	Slide	Rise	Tilt	Roll	Twist
1 CG/CG	-0.08	-0.01	3.15	-2.85	5.87	34.52
2 GC/GC	0.56	0.45	3.68	2.53	-7.98	42.43
3 CG/CG	-0.41	0.68	2.97	2.83	10.01	26.60
4 GA/TC	0.13	-0.19	3.38	0.38	2.13	38.15
5 AT/AT	0.51	-0.69	3.29	0.95	-1.36	32.63
6 TA/TA	0.33	-0.20	3.38	1.77	2.78	35.56
7 AT/AT	0.07	-0.63	3.28	2.20	-2.12	32.08
8 TC/GA	-0.41	-0.20	3.46	0.65	1.00	40.40
9 CG/CG	0.48	0.89	2.99	-4.06	5.13	26.77
10 GC/GC	-1.11	0.62	3.48	-1.98	-12.01	43.14
11 CG/CG	0.22	0.32	3.22	-1.96	5.46	32.92
ave.	0.03	0.09	3.30	0.04	0.81	35.02

Table S9: Helical Parameters for ATATAT-DB1476

Local base-pair parameters

bp	Shear	Stretch	Stagger	Buckle	Propeller	Opening
1 G-C	-0.21	-0.06	0.09	0.02	-6.74	0.07
2 C-G	0.20	-0.10	0.48	-10.86	-8.98	-2.57
3 T-A	0.03	-0.18	0.24	-6.06	-15.46	1.07
4 G-C	-0.16	-0.22	-0.09	-7.10	-3.43	-2.49
5 G-C	-0.03	-0.22	-0.12	5.90	-10.64	1.70
6 A-T	0.11	-0.05	0.02	3.70	-13.51	-0.35
7 T-A	0.08	-0.17	-0.05	8.78	-8.87	1.57
8 A-T	0.09	-0.13	0.02	-1.07	-12.16	4.63
9 T-A	-0.21	-0.19	-0.13	1.51	-13.23	4.88
10 A-T	-0.12	-0.07	-0.02	-5.69	-9.87	3.17
11 T-A	-0.28	-0.03	0.01	-3.24	-15.80	-0.60
12 C-G	0.02	-0.05	-0.13	-5.07	-12.48	3.77
13 C-G	0.25	-0.23	-0.16	6.01	-2.43	-2.47
14 A-T	0.04	-0.14	0.39	9.03	-15.44	2.51
15 G-C	-0.18	-0.19	0.53	9.85	-10.47	-2.65
16 C-G	0.20	-0.09	0.05	0.01	-8.86	0.64
~~						
ave.	-0.01	-0.13	0.07	0.36	-10.52	0.81

Local base-pair step parameters

step	Shift	Slide	Rise	Tilt	Roll	Twist
1 GC/GC	-0.93	-0.60	3.49	-3.88	1.12	32.50
2 CT/AG	-0.47	-0.16	3.22	2.50	1.85	28.50
3 TG/CA	0.40	1.20	3.41	3.36	-0.29	43.61
4 GG/CC	0.87	0.13	3.17	-0.07	6.19	21.73
5 GA/TC	-1.05	0.25	3.32	-4.52	-0.96	41.12
6 AT/AT	0.47	-0.37	3.22	0.08	-2.39	29.89
7 TA/TA	0.09	0.36	3.53	-0.01	-0.83	42.13
8 AT/AT	-0.00	-0.60	3.23	1.26	0.48	29.88
9 TA/TA	-0.03	0.40	3.51	-1.31	-0.28	43.10

10 AT/AT	-0.48	-0.46	3.21	-0.21	-1.96	28.92
11 TC/GA	1.15	0.20	3.28	4.37	-1.61	41.49
12 CC/GG	-0.89	0.15	3.23	1.24	6.52	20.74
13 CA/TG	-0.29	1.27	3.31	-3.90	-0.88	44.30
14 AG/CT	0.47	-0.18	3.26	-1.46	2.27	28.69
15 GC/GC	1.18	-0.62	3.46	6.09	-0.23	32.35
~~						
ave.	0.03	0.07	3.32	0.24	0.60	33.93

Table S10: Helical Parameters for A4-DB1476

Local base-pair parameters

bp	Shear	Stretch	Stagger	Buckle	Propeller	Opening
1 C-G	0.22	-0.17	0.28	1.20	-11.93	-1.59
2 G-C	-0.16	-0.23	0.61	10.07	-10.92	-3.25
3 C-G	0.20	-0.24	0.27	-6.67	-5.33	0.45
4 G-C	-0.19	-0.12	0.12	10.89	-7.25	0.25
5 A-T	0.39	-0.10	0.03	6.30	-19.47	2.94
6 A-T	0.26	-0.28	-0.03	0.88	-20.58	3.83
7 A-T	0.10	-0.23	-0.01	1.01	-20.54	5.48
8 A-T	0.20	-0.19	-0.03	-2.87	-15.97	5.12
9 C-G	0.19	-0.07	0.11	-11.13	-8.80	-1.28
10 G-C	-0.05	-0.15	0.34	11.95	-3.86	2.16
11 C-G	0.08	-0.23	0.34	3.23	-21.63	-3.90
12 G-C	-0.15	-0.17	0.48	6.33	-6.79	-2.95
~~						
ave.	0.09	-0.18	0.21	2.60	-12.76	0.60

Local base-pair step parameters

step	Shift	Slide	Rise	Tilt	Roll	Twist
1 CG/CG	-0.13	-0.04	3.04	-2.92	5.09	34.51
2 GC/GC	0.64	0.37	3.75	3.24	-7.41	42.47
3 CG/CG	-0.26	0.64	2.99	2.61	8.92	27.37
4 GA/TC	0.12	-0.13	3.39	1.20	3.56	37.49

5 AA/TT	0.12	-0.35	3.42	1.24	0.13	35.57
6 AA/TT	0.19	-0.25	3.18	1.18	1.59	32.40
7 AA/TT	-0.03	-0.23	3.32	0.53	0.87	36.06
8 AC/GT	-0.53	-0.39	3.55	-0.67	-1.82	37.16
9 CG/CG	0.51	0.84	2.88	-2.97	3.49	27.40
10 GC/GC	-1.08	0.56	3.59	-1.82	-10.18	41.65
11 CG/CG	0.21	0.34	3.19	-0.69	4.59	34.26
~~						
ave.	-0.02	0.12	3.30	0.08	0.80	35.12

Table S11: Helical Parameters for A6-DB1476-I

Local base-pair parameters

bp	Shear	Stretch	Stagger	Buckle	Propeller	Opening
1 C-G	-0.07	-0.23	0.28	-3.22	-7.47	-3.99
2 G-C	-0.28	-0.30	0.20	1.59	-19.80	-2.80
3 C-G	0.39	-0.31	-0.12	-0.96	-5.41	1.70
4 A-T	-0.08	-0.14	0.05	13.88	-12.92	1.65
5 A-T	0.09	-0.12	0.31	10.41	-20.37	-0.06
6 A-T	0.05	-0.26	-0.03	0.58	-21.81	4.36
7 A-T	1.61	-0.23	-0.05	-9.50	-24.52	9.76
8 A-T	0.74	-0.37	0.14	-12.03	-14.71	6.10
9 A-T	1.14	0.13	-0.66	-9.16	-12.06	-2.82
10 G-C	-0.01	-0.04	0.57	8.35	-1.68	3.31
11 C-G	0.21	-0.35	0.42	-0.11	-24.40	-3.36
12 G-C	0.18	-0.15	0.83	10.65	1.00	-5.08
~~						
ave.	0.33	-0.20	0.16	0.87	-13.68	0.73

Local base-pair step parameters

step	Shift	Slide	Rise	Tilt	Roll	Twist
1 CG/CG	-0.01	0.01	3.20	0.58	6.87	36.26
2 GC/GC	0.93	0.43	3.24	4.72	-7.63	42.89
3 CA/TG	-0.37	0.82	3.13	1.90	6.00	24.17

4 AA/TT	-0.22	0.20	3.25	-3.04	-1.82	38.02
5 AA/TT	0.35	-0.27	3.43	4.11	5.06	37.46
6 AA/TT	0.05	0.11	3.54	0.52	3.86	40.62
7 AA/TT	-0.65	0.04	3.13	-3.00	2.41	32.43
8 AA/TT	-0.99	0.22	3.23	6.17	-5.08	39.96
9 AG/CT	0.74	0.26	2.81	-11.16	6.28	22.70
10 GC/GC	-1.16	0.53	3.55	-0.29	-10.84	42.31
11 CG/CG	0.44	0.22	3.11	-2.14	-3.56	36.61
~~						
ave.	-0.08	0.23	3.24	-0.15	0.14	35.77

Table S12: Helical Parameters for A6-DB1476-II

Local base-pair parameters

bp	Shear	Stretch	Stagger	Buckle	Propeller	Opening
1 C-G	-0.08	-0.27	0.30	-3.82	-9.48	-3.97
2 G-C	-0.36	-0.29	0.28	2.92	-17.70	-2.97
3 C-G	0.38	-0.30	-0.12	-0.94	-3.85	0.82
4 A-T	-0.13	-0.13	0.09	14.19	-11.09	2.60
5 A-T	0.05	-0.08	0.20	11.27	-21.35	1.22
6 A-T	0.01	-0.23	-0.04	1.83	-20.96	4.62
7 A-T	1.57	-0.25	-0.13	-8.51	-25.17	10.76
8 A-T	0.69	-0.35	0.12	-12.05	-14.75	6.53
9 A-T	1.19	0.06	-0.62	-7.26	-12.13	-4.52
10 G-C	-0.07	-0.07	0.57	9.89	-2.58	3.71
11 C-G	0.28	-0.33	0.42	-0.03	-23.31	-3.48
12 G-C	0.15	-0.20	0.93	12.10	2.53	-6.49
~~						
ave.	0.31	-0.20	0.17	1.63	-13.32	0.74

Local base-pair step parameters

step	Shift	Slide	Rise	Tilt	Roll	Twist
1 CG/CG	-0.04	0.01	3.15	-0.46	5.67	35.78
2 GC/GC	0.89	0.39	3.29	5.18	-7.34	43.13

3 CA/TG	-0.32	0.79	3.11	1.58	6.40	24.03
4 AA/TT	-0.31	0.28	3.26	-2.51	-1.36	37.94
5 AA/TT	0.34	-0.23	3.42	3.08	3.86	37.42
6 AA/TT	0.11	0.20	3.56	1.19	4.70	40.98
7 AA/TT	-0.71	0.01	3.13	-3.63	3.00	32.04
8 AA/TT	-1.08	0.24	3.16	5.68	-5.27	40.58
9 AG/CT	0.83	0.26	2.85	-10.40	5.53	21.88
10 GC/GC	-1.17	0.55	3.58	-0.57	-10.30	43.28
11 CG/CG	0.44	0.16	3.03	-3.09	-3.53	36.42

~~~~~~

ave.	-0.09	0.24	3.23	-0.36	0.12	35.77

Table S13: Helical Parameters for AATT_NAT

Local base-pair parameters

bp	Shear	Stretch	Stagger	Buckle	Propeller	Opening
1 C-G	0.30	-0.16	0.08	5.42	-14.68	0.01
2 G-C	-0.24	-0.23	0.40	6.42	-12.97	-2.20
3 C-G	0.14	-0.19	0.24	-4.80	-5.98	0.18
4 G-C	-0.25	-0.08	0.08	11.58	-7.67	1.34
5 A-T	0.02	-0.09	-0.04	6.09	-13.77	2.69
6 A-T	0.02	-0.15	0.08	1.92	-15.60	4.26
7 T-A	-0.05	-0.14	0.09	-0.02	-15.73	3.69
8 T-A	-0.10	-0.16	-0.02	-0.97	-13.38	4.11
9 C-G	0.21	-0.15	0.09	-12.88	-7.18	-1.90
10 G-C	-0.10	-0.16	0.27	5.83	-8.31	2.21
11 C-G	0.20	-0.24	0.27	0.23	-17.12	-2.78
12 G-C	-0.21	-0.05	0.36	3.67	-6.55	-1.63

~~~~~~

ave.	-0.01	-0.15	0.16	1.87	-11.58	0.83

Local base-pair step parameters

step	Shift	Slide	Rise	Tilt	Roll	Twist
1 CG/CG	-0.01	0.09	3.26	-2.56	6.95	33.82

2 GC/GC	0.56	0.57	3.61	1.42	-7.00	42.96
3 CG/CG	-0.26	0.60	3.02	1.40	9.10	26.09
4 GA/TC	-0.16	-0.19	3.38	0.16	3.15	36.73
5 AA/TT	0.09	-0.44	3.31	-1.03	0.30	36.19
6 AT/AT	0.07	-0.63	3.26	0.29	-1.77	32.41
7 TT/AA	-0.02	-0.46	3.22	1.33	-1.61	34.34
8 TC/GA	-0.24	-0.29	3.54	0.11	-2.30	42.34
9 CG/CG	0.61	0.67	2.98	-2.54	4.50	25.83
10 GC/GC	-1.24	0.49	3.47	-3.30	-9.88	42.65
11 CG/CG	-0.12	0.24	3.17	-1.30	5.29	32.91

~~~~~~

ave.	-0.07	0.06	3.29	-0.55	0.61	35.12

Table S14: Helical Parameters for AAATTT_NAT

Local base-pair parameters

bp	Shear	Stretch	Stagger	Buckle	Propeller	Opening
1 C-G	0.17	-0.16	0.03	2.91	-3.43	-2.77
2 G-C	-0.13	-0.09	0.23	-3.37	-5.37	-4.19
3 C-G	0.16	-0.17	-0.01	-0.08	-7.24	0.01
4 A-T	-0.02	-0.06	-0.10	9.76	-15.31	4.82
5 A-T	-0.05	-0.21	0.25	12.87	-18.45	1.15
6 A-T	-0.01	-0.19	0.31	4.58	-17.55	4.95
7 T-A	0.09	-0.16	0.28	-2.33	-20.13	4.58
8 T-A	-0.20	-0.12	0.12	-7.40	-19.74	5.53
9 T-A	-0.02	-0.07	0.07	-12.06	-14.48	-0.49
10 G-C	-0.29	-0.18	0.35	9.84	-4.97	2.82
11 C-G	0.08	-0.22	0.32	3.05	-18.01	-2.00
12 G-C	-0.12	-0.05	0.23	8.49	-5.53	0.39

~~~~~~

ave.	-0.03	-0.14	0.17	2.19	-12.52	1.23

Local base-pair step parameters

step	Shift	Slide	Rise	Tilt	Roll	Twist

1 CG/CG	-0.83	-0.24	3.55	-3.95	-6.48	35.28
2 GC/GC	0.95	0.39	3.22	3.89	-0.55	39.71
3 CA/TG	-0.57	0.82	3.30	2.83	12.12	26.57
4 AA/TT	-0.04	-0.04	3.12	-1.52	-1.82	34.72
5 AA/TT	0.23	-0.36	3.33	-1.87	0.57	39.53
6 AT/AT	-0.09	-0.69	3.36	0.44	-1.25	32.70
7 TT/AA	-0.18	-0.33	3.27	1.81	-2.18	35.51
8 TT/AA	-0.19	-0.08	3.30	1.63	-3.62	41.67
9 TG/CA	0.87	0.85	2.90	-3.04	1.94	27.62
10 GC/GC	-1.36	0.55	3.50	-2.27	-7.65	42.82
11 CG/CG	0.97	0.55	3.27	2.57	2.42	34.23

~~~~~~

ave.	-0.02	0.13	3.28	0.05	-0.59	35.49

Table S15: Helical Parameters for ATAT_NAT

Local base-pair parameters

bp	Shear	Stretch	Stagger	Buckle	Propeller	Opening
1 C-G	0.28	-0.09	0.38	2.47	-16.21	0.13
2 G-C	-0.14	-0.11	0.34	5.11	-15.37	-0.07
3 C-G	0.06	-0.19	0.24	-6.98	-7.23	-0.63
4 G-C	-0.41	-0.04	-0.07	10.53	-10.96	2.73
5 A-T	0.11	-0.12	0.24	9.71	-14.44	-0.27
6 T-A	-0.05	-0.13	-0.07	7.89	-13.43	5.32
7 A-T	0.04	-0.09	0.05	-5.18	-12.29	1.03
8 T-A	-0.06	-0.16	0.19	-2.86	-12.04	4.27
9 C-G	0.18	-0.11	0.12	-10.57	-11.11	-0.81
10 G-C	0.00	-0.15	0.38	10.82	-9.06	1.13
11 C-G	0.03	-0.21	0.65	-6.19	-14.62	-3.96
12 G-C	-0.27	-0.07	0.31	6.57	-4.33	0.05

~~~~~~

ave.	-0.02	-0.12	0.23	1.78	-11.76	0.74

Local base-pair step parameters

step	Shift	Slide	Rise	Tilt	Roll	Twist

1 CG/CG	-0.06	0.04	3.24	-0.65	7.37	34.21
2 GC/GC	0.48	0.51	3.59	0.69	-9.41	42.53
3 CG/CG	-0.28	0.66	3.02	3.49	11.21	26.64
4 GA/TC	-0.15	-0.20	3.22	-2.64	2.25	36.39
5 AT/AT	0.51	-0.80	3.29	2.71	-0.19	32.83
6 TA/TA	0.01	-0.60	3.55	-0.48	1.10	36.78
7 AT/AT	0.07	-0.77	3.23	-0.17	-4.69	32.25
8 TC/GA	-0.30	-0.30	3.41	2.05	-3.14	41.63
9 CG/CG	0.56	0.72	2.85	-3.44	4.58	27.26
10 GC/GC	-1.16	0.36	3.75	-6.31	-10.91	40.92
11 CG/CG	0.04	0.19	2.96	2.13	3.19	33.85

~~~~~~

ave.	-0.02	-0.02	3.28	-0.24	0.12	35.02

Table S16: Helical Parameters for ATATAT_NAT

Local base-pair parameters

bp	Shear	Stretch	Stagger	Buckle	Propeller	Opening
1 G-C	-0.29	-0.04	0.05	-0.51	-7.80	1.50
2 C-G	0.19	-0.16	0.40	-6.83	-10.20	-2.29
3 T-A	-0.02	-0.08	0.17	-3.63	-15.61	-0.08
4 G-C	-0.20	-0.17	-0.20	-7.68	-1.31	-1.94
5 G-C	-0.10	-0.14	-0.03	7.02	-9.58	1.42
6 A-T	0.03	-0.14	0.07	6.06	-11.55	1.44
7 T-A	0.04	-0.14	0.08	8.43	-9.97	2.66
8 A-T	0.01	-0.08	0.17	-1.85	-10.39	4.86
9 T-A	-0.01	-0.08	0.17	1.85	-10.39	4.86
10 A-T	-0.04	-0.14	0.08	-8.43	-9.97	2.66
11 T-A	-0.03	-0.14	0.07	-6.06	-11.55	1.44
12 C-G	0.10	-0.14	-0.03	-7.02	-9.58	1.42
13 C-G	0.20	-0.17	-0.20	7.68	-1.31	-1.94
14 A-T	0.02	-0.08	0.17	3.63	-15.61	-0.08
15 G-C	-0.19	-0.16	0.40	6.83	-10.20	-2.29
16 C-G	0.29	-0.04	0.05	0.51	-7.80	1.50

~~~~~~

ave.	0.00	-0.12	0.09	0.00	-9.55	0.95

Local base-pair step parameters

step	Shift	Slide	Rise	Tilt	Roll	Twist
1 GC/GC	-1.02	-0.54	3.40	-4.16	-0.40	33.46
2 CT/AG	-0.52	-0.02	3.22	2.16	1.84	29.14
3 TG/CA	0.60	1.04	3.47	4.12	-0.03	43.12
4 GG/CC	0.87	-0.02	3.13	-2.16	5.56	19.44
5 GA/TC	-0.91	0.20	3.29	-3.00	-1.48	41.44
6 AT/AT	0.40	-0.52	3.24	-0.34	-2.20	30.91
7 TA/TA	-0.04	-0.11	3.47	-0.77	-1.28	41.50
8 AT/AT	0.00	-0.49	3.19	0.00	-5.10	31.13
9 TA/TA	0.04	-0.11	3.47	0.77	-1.28	41.50
10 AT/AT	-0.40	-0.52	3.24	0.34	-2.20	30.91
11 TC/GA	0.91	0.20	3.29	3.00	-1.48	41.44
12 CC/GG	-0.87	-0.02	3.13	2.16	5.56	19.44
13 CA/TG	-0.60	1.04	3.47	-4.12	-0.03	43.12
14 AG/CT	0.52	-0.02	3.22	-2.16	1.84	29.14
15 GC/GC	1.02	-0.54	3.40	4.16	-0.40	33.46

~~~~~~

ave.	0.00	-0.03	3.31	-0.00	-0.07	33.95

Table S17: Helical Parameters for A6_NAT

Local base-pair parameters

bp	Shear	Stretch	Stagger	Buckle	Propeller	Opening
1 C-G	0.63	-0.31	0.07	5.81	-16.07	2.97
2 G-C	-0.60	-0.18	0.06	-0.53	-9.42	-0.93
3 C-G	0.03	0.09	-0.01	-3.87	-6.75	5.81
4 A-T	0.58	-0.07	0.10	7.12	-12.60	2.33
5 A-T	0.87	-0.24	0.36	12.42	-15.32	1.98
6 A-T	0.61	-0.26	0.22	7.18	-23.01	2.29
7 A-T	0.74	-0.19	0.14	-2.71	-14.98	2.29
8 A-T	0.82	-0.23	0.12	-6.52	-19.60	-1.73
~~~~~~

9 A-T	0.50	0.43	-0.21	-11.06	-14.25	2.75
10 G-C	0.00	0.01	0.57	11.18	-6.38	5.73
11 C-G	-0.20	-0.21	-0.10	4.00	-15.22	-4.68
12 G-C	0.39	-0.01	0.21	0.95	-10.50	-4.80

~~~~~~

ave.	0.37	-0.10	0.13	2.00	-13.67	1.17

Local base-pair step parameters

step	Shift	Slide	Rise	Tilt	Roll	Twist
1 CG/CG	-0.20	0.17	3.58	-0.55	-0.32	32.86
2 GC/GC	0.93	0.35	3.35	2.82	-0.11	39.93
3 CA/TG	-0.71	0.49	3.22	0.34	7.58	32.63
4 AA/TT	-0.09	-0.19	3.08	-2.46	-3.71	35.58
5 AA/TT	-0.05	-0.23	3.30	3.37	-1.67	39.36
6 AA/TT	-0.20	-0.43	3.36	0.79	-0.71	34.83
7 AA/TT	-0.08	-0.49	3.39	2.27	-2.04	36.31
8 AA/TT	0.04	-0.27	3.29	4.98	-4.44	35.91
9 AG/CT	0.79	0.46	2.81	-7.46	6.49	26.16
10 GC/GC	-1.57	0.19	3.41	0.95	-7.41	38.89
11 CG/CG	0.12	0.43	3.53	-1.49	3.53	40.14

~~~~~~

ave.	-0.09	0.04	3.30	0.32	-0.26	35.69

Table S18: Molecular structure with specific atom (.frcmod file) types used for the DB1476 molecule

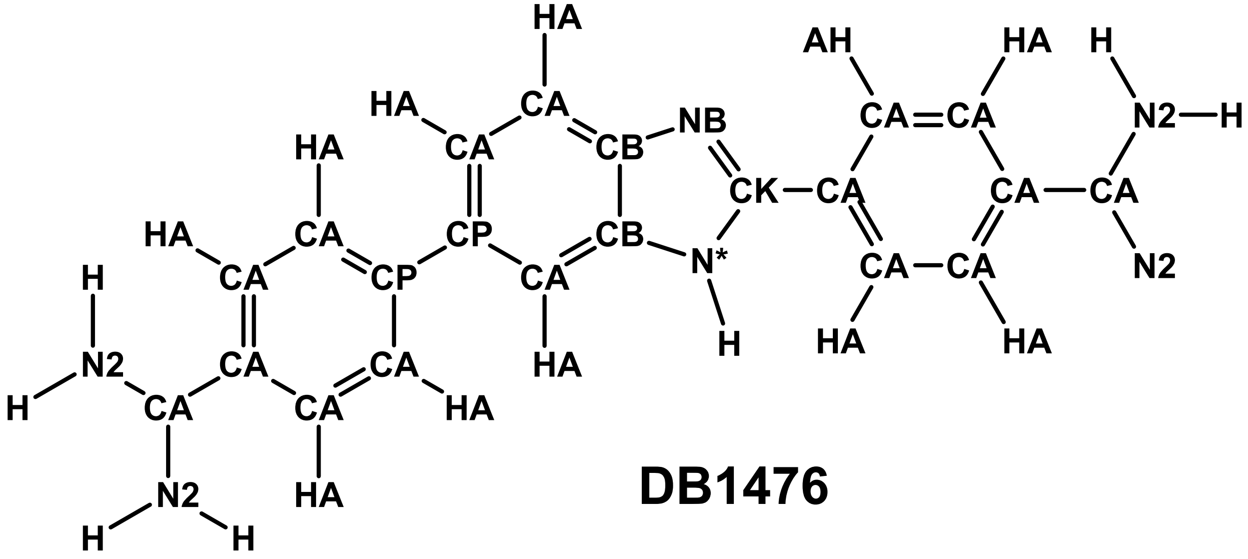

remark goes here
MASS

N2 14.01	0.530	parm99
CA 12.01	0.360	parm99
CB 12.01	0.360	parm99
CK 12.01	0.360	parm99
HA 1.008	0.167	parm99
H 1.008	0.161	parm99
N* 14.01	0.530	parm99
NB 14.01	0.530	parm99
CP 12.01	0.360	gaff(cp)

BOND

CA-CA	469.0	1.400	parm99
CA-CB	469.0	1.404	parm99
CB-CB	520.0	1.370	parm99
CB-N*	436.0	1.374	parm99
CB-NB	414.0	1.391	parm99
CA-HA	367.0	1.080	parm99
CK-N*	440.0	1.371	parm99
CK-NB	529.0	1.304	parm99
CA-N2	481.0	1.340	parm99
H -N2	434.0	1.010	parm99 for plain unmethylated bases ADE,CYT,GUA,ARG
H -N*	434.0	1.010	parm99 for plain unmethylated bases ADE,CYT,GUA,ARG
CK-CA	469.0	1.475	FORCE CONTST: parm99, CA-CB: length-Gaussian
CP-CP	346.5	1.4900	GAFF

CP-CA 466.1 1.3950 Gaff

ANGLE

CA-CA-CA	63.0	120.00	parm99
CA-CA-CB	63.0	120.00	parm99
CA-CA-HA	50.0	120.00	parm99
CA-CB-CB	63.0	117.30	parm99
CB-CA-HA	50.0	120.00	parm99
CA-CB-NB	70.0	132.40	parm99
CA-CB-N*	70.0	132.40	parm99 CA-CB-NB parm99
CB-CB-N*	70.0	106.20	parm99
CB-CB-NB	70.0	110.40	parm99
N*-CK-NB	70.0	113.90	parm99
CB-NB-CK	70.0	103.80	parm99
CB-N*-CK	70.0	105.40	parm99
CK-N*-H	50.0	128.80	for unmethylated n.a. bases,chngd bsd NMA nmodes
CB-N*-H	50.0	125.80	for unmethylated n.a. bases,chngd bsd NMA nmodes
CA-CA-N2	70.0	119.99	parm99, CM-CA-N2, Gaussian-angle
N2-CA-N2	70.0	120.00	parm99
H -N2-H	35.0	120.00	parm99
CA-N2-H	50.0	120.00	parm99
CA-CK-NB	67.790	120.310	gaff SOURCE4 ca-cc-nc 46 0.6711
CK-CA-CA	65.990	120.100	GAFF ca-ca-cc SOURCE3 1030.3451
N*-CK-CA	67.790	120.310	gaff ca-cc-nc 46 0.6711
CA-CP-CP	64.140	121.140	GAFF
CP-CA-HA	48.030	121.080	GAFF
CA-CA-CP	67.240	119.070	GAFF
CA-CP-CA	67.250	118.330	GAFF
CP-CA-CB	67.240	119.070	GAFF ca-ca-cp

DIHE

N2-CA-N2-H	4	9.60	180.0	2.0	parm 99, X -CA-N2-X
H -N2-CA-CA	4	9.60	180.0	2.0	parm 99, X -CA-N2-X
N2-CA-CA-CA	4	-3.118	0.000	-2.0	DB921
N2-CA-CA-CA	4	0.789	327.0	-4.0	DB921
N2-CA-CA-CA	4	0.609	90.000	1.0	DB921
CA-CA-CA-CA	4	14.50	180.0	2.0	parm99, X -CA-CA-X
CA-CA-CA-HA	4	14.50	180.0	2.0	parm99, X -CA-CA-X
HA-CA-CA-HA	4	14.50	180.0	2.0	parm99, X -CA-CA-X
CB-CA-CA-HA	4	14.50	180.0	2.0	parm99, X -CA-CA-X
CA-CA-CB-CB	4	14.00	180.0	2.0	parm99, X -CA-CB-X
HA-CA-CB-N*	4	14.00	180.0	2.0	parm99, X -CA-CB-X
HA-CA-CB-NB	4	14.00	180.0	2.0	parm99, X -CA-CB-X
HA-CA-CB-CB	4	14.00	180.0	2.0	parm99, X -CA-CB-X
CB-CB-N*-CK	4	6.60	180.0	2.0	parm99, X -CB-N*-X
CB-CB-N*-H	4	6.60	180.0	2.0	pram99, X -CB-N*-X

CA-CB-N*-H	4	6.60	180.0	2.0	pram99, X -CB-N*-X
CA-CB-CB-NB	4	21.80	180.0	2.0	parm99, X -CB-CB-X
CA-CB-CB-N*	4	21.80	180.0	2.0	parm99, X -CB-CB-X
CA-CA-CB-NB	4	14.00	180.0	2.	intrpol.bsd.on C6H6, X -CA-CB-X
CA-CB-NB-CK	2	5.10	180.0	2.0	parm99, X -CB-NB-X
CA-CB-N*-CK	4	6.60	180.0	2.	JCC,7,(1986),230, X -CB-N*-X
NB-CK-N*-CB	4	6.80	180.0	2.0	parm99, X -CK-N*-X
N*-CK-NB-CB	2	20.00	180.0	2.0	parm99, X -CK-NB-X
CB-N*-CK-CA	4	6.80	180.0	2.0	parm99, X -CK-N*-X
NB-CK-CA-CA	4	3.1	180.0	-2.0	DB921 for NB-CK-CA-CA
NB-CK-CA-CA	4	-0.6	180.0	-4.0	DB921 for NB-CK-CA-CA
NB-CK-CA-CA	4	-0.7	360.0	1.0	DB921 for NB-CK-CA-CA
N*-CK-CA-CA	4	-0.6	180.0	-4.0	DB921
N*-CK-CA-CA	4	3.1	180.0	-2.0	DB921
N*-CK-CA-CA	4	-0.7	360.0	1.0	DB921
H-N*-CK-CA	4	6.80	180.0	2.0	PARM99(X-CK-N*-X)
NB-CK-N*-H	4	6.800	180.0	2.0	PARM99(X-CK-N*-X)
CK-CA-CA-CA	4	14.50	180.0	2.0	parm99, X -CA-CA-X
CK-CA-CA-HA	4	14.50	180.0	2.0	parm99, X -CA-CA-X
N*-CB-CB-NB	4	21.80	180.0	2.0	PARM99 X -CB-CB-X
CA-CB-CB-CA	4	21.80	180.0	2.0	PARM99 X -CB-CB-X
CP-CA-CB-N*	4	14.00	180.0	2.0	PARM99 X -CA-CB-X
CB-CB-NB-CK	2	5.10	180.0	2.0	PARM99 X -CB-NB-X
CP-CP-CA-HA	4	14.5	180.0	2.0	DB921
CP-CP-CA-CA	4	14.5	180.0	2.0	DB921
CP-CA-CA-HA	4	14.5	180.0	2.0	DB921
CP-CA-CA-CA	4	14.5	180.0	2.0	DB921
CA-CP-CP-CA	4	4.000	180.000	2.000	GAFF, X -cp-cp-X
CA-CP-CA-CA	4	14.5	180.0	2.0	DB921
CA-CP-CA-HA	4	14.5	180.0	2.0	DB921
CP-CA-CA-CB	4	14.5	180.0	2.0	DB921
CP-CA-CB-CB	4	14.00	180.0	2.0	PARM99 X -CA-CB-X
CA-CP-CA-CB	4	14.500	180.000	2.000	GAFF, X -ca-cp-X
CP-CP-CA-CB	4	14.5	180.0	2.0	DB921

IMPROPER

CA-CA-CA-CP	1.1	180.0	2.0	Using default value
CA-CA-CA-HA	1.1	180.0	2.0	General improper torsional angle (2 general atom types)
CA-CA-CP-CA	1.1	180.0	2.0	Using default value
CP-CA-CA-HA	1.1	180.0	2.0	Using default value
CP-CA-CA-HA	1.1	180.0	2.0	Using default value
CA-CA-CA-CA	1.1	180.0	2.0	Using default value
CA-N2-CA-N2	1.1	180.0	2.0	Using default value

CA-CB-CB-NB	1.1	180.0	2.0	Using default value
CA-CB-CB-NA	1.1	180.0	2.0	Using default value
CB-CK-N*-H	1.1	180.0	2.0	General improper torsional angle (2 general atom types)
CA-N*-CK-NB	1.1	180.0	2.0	Using default value
CK-CA-CA-CA	1.1	180.0	2.0	Using default value

NONBON

H	0.6000	0.0157	parm99
HA	1.4590	0.0150	parm99
CA	1.9080	0.0860	parm99 (C*)
CB	1.9080	0.0860	parm99 (C*)
CK	1.9080	0.0860	parm99 (C*)
N*	1.8240	0.1700	parm99 (N)
NB	1.8240	0.1700	parm99 (N)
N2	1.8240	0.1700	parm99 (N)
CP	1.9080	0.0860	PARM99
